# A dual-fMRI investigation of interpersonal emotion regulation: Predicting strategy selection and implementation success from effective brain connectivity

**DOI:** 10.1162/IMAG.a.1360

**Published:** 2026-09-11

**Authors:** Nicola Ngombe, Kristína Czekóová, Martin Gajdoš, Klaus Kessler, Milan Brázdil, Simone Shamay-Tsoory, Daniel Joel Shaw

**Affiliations:** Cajal Neuroscience Centre, Consejo Superior de Investigaciones Científicas, Madrid, Spain; Institute of Psychology, Czech Academy of Sciences, Brno, Czechia; Behavioural and Social Neuroscience Research Group, Central European Institute of Technology (CEITEC), Masaryk University, Brno, Czechia; Multi-modal and Functional Neuroimaging Research Group, Central European Institute of Technology (CEITEC), Masaryk University, Brno, Czechia; School of Psychology, University College Dublin, Dublin, Ireland; School of Psychological Science, University of Haifa, Haifa, Israel; School of Psychology, Aston University, Birmingham, United Kingdom

**Keywords:** inter-personal emotion regulation, strategy selection, implementation, second-person neuroscience, functional magnetic resonance imagining, behavioural dynamic causal modelling

## Abstract

Inter-personal emotion regulation (ER) is the social process whereby one individual (a “Regulator”) assists another (the “Target”) in regulating their emotional state. Despite the importance of inter-personal ER for maintaining well-being, very little is known about the neurocognitive mechanisms that support this process. In the present study, we performed functional magnetic resonance imaging on 23 pairs of Regulators and Targets whilst they engaged in a novel task designed to capture the two stages of inter-personal ER: the Regulator’s selection of an ER strategy to recommend and its subsequent implementation by an affiliate Target. This paradigm allowed us to investigate the brain systems supporting both stages of the inter-personal process and identify personality characteristics that might influence the inter-personal dynamic through these neurocognitive mechanisms. Results revealed largely overlapping patterns of brain response during Regulators’ selections and Targets’ implementation of ER strategies, encompassing medial and lateral prefrontal and temporo-parietal cortices. Moreover, by applying behavioural Dynamic Causal Modelling to the behavioural and brain data acquired during the task, we identified patterns of effective connectivity among these brain regions from which we could accurately estimate Regulators’ selections and their effectiveness in down-regulating Targets’ emotional responses. Lastly, certain network connections and the effectiveness of Regulators’ selections in down-regulating Targets’ emotion states were associated with two dissociable styles of self-directed (intra-personal) ER: failure- and decision-related action control.

## 1 Introduction

Effective emotion regulation (ER) is crucial for maintaining well-being (Chen et al., 2025), as evidenced by the apparent ubiquity of ER difficulties across psychopathologies (Sheppes et al., 2015). Decades of research has characterised the various cognitive and associated brain mechanisms supporting intra-personal ER, the process through which we regulate our own emotional states without the involvement of others (Morawetz et al., 2017, 2020). Although this has provided invaluable insights into the neurocognitive processes underpinning functional and dysfunctional self-directed ER (Gross, 2015; McTeague et al., 2020; Morawetz et al., 2020), ER often occurs through social interactions (Petrova & Gross, 2023); we try to support others in regulating their emotions (Nozaki & Mikolajczak, 2020) and follow the guidance of those around us when attempting to regulate our own emotional responses (Zaki & Williams, 2013). Importantly, both forms of inter-personal ER have been shown to promote well-being by reducing stress and alleviating negative affectivity in those involved (Cohen & Arbel, 2020; Guendelman et al., 2022). Although recent research has begun to reveal factors that influence the effectiveness of inter-personal ER, such as the personality characteristics of participating individuals (Naor-Ziv et al., 2023) and the closeness of their interpersonal relationship (Tanna & MacCann, 2023), little is known about the underpinning neurocognitive mechanisms. This is true particularly for the process of regulating another person’s emotions, despite its relatively higher occurrence in everyday social interactions (Liu et al., 2021; Tran et al., 2023). Given the importance of inter-personal ER for our own well-being and that of those around us (Heiland et al., 2026), the aim of the present study was to elucidate the neurocognitive mechanisms that support this social cognitive process.

Intra-personal ER involves three distinct stages (Gross, 2015): individuals must first recognise a discrepancy between their current/expected and desired emotional state (identification), then select a strategy to regulate their emotion to achieve the desired state (selection) and subsequently implement the selected strategy (implementation). Different strategies target different parts of an emotional response—either before or after it has been generated (antecedent- or response-focused, respectively). During inter-personal ER, a “Regulator” attempts to modify the emotional response of a “Target”. As with intra-personal ER, this can occur at different parts of the emotion-generative process (Gross, 2015); for example, Regulators can modulate a Target’s negative emotional response before it has developed fully by advising them not to attend to negative emotion-evoking situations or by guiding them in reappraising such situations to have less negative meaning. Regardless of when in the emotion-generative process it occurs, however, the effectiveness of inter-personal ER relies upon the Regulator accurately identifying a discrepancy between the Target’s current (or future) emotional state and an extrinsic ER goal, then select an ER strategy to recommend that will enable the Target to achieve that goal, and the Target must subsequently implement the recommended strategy (Nozaki & Mikolajczak, 2020; Reeck et al., 2016). Any neurocognitive description of inter-personal ER must, therefore, capture each stage of the process in both the Regulator and Target.

In a theoretical model proposed by Reeck et al. (2016), three interconnected neural systems are hypothesised to interact in systematic ways during these stages of inter-personal ER. In Regulators, a social cognitive system comprising posterior temporal and temporo-parietal cortex guides inferences about the Target’s emotional state and simulates the effects of different ER strategies. This social cognitive system then steers a cognitive control system, consisting of the ventro- and dorso-lateral prefrontal cortex, in the selection of an ER strategy to recommend. In Targets, the model proposes that an emotion-generative system comprising the amygdala and ventral striatum is regulated by the cognitive control system under the direction of the social cognitive system that processes the Regulator’s recommendations. Although studies provide empirical support for the co-involvement of these three brain systems during inter-personal ER in both Regulators (Guendelman et al., 2022; Hallam et al., 2014) and Targets (Morawetz et al., 2021; Zhang, Qiu, Tang, & Sun, 2023), they have focused only on the final implementation stage. Such similarity might, therefore, be indicative of shared processes, such as simulation and empathic sharing (Kozakevich Arbel et al., 2021), and a simultaneous (implicit) down-regulation of an emotional state in Regulators whilst they assist Targets (emotional co-regulation; see Guendelman et al., 2022). It remains to be seen if or how these systems interact during the earlier stage of Regulators’ ER strategy selection, and how this compares with the brain systems supporting the subsequent implementation by Targets. The present study addressed this knowledge gap by performing the first assessment of brain connectivity during both stages of inter-personal ER.

Elucidating patterns of brain connectivity that support inter-personal ER as it occurs in the real world requires Regulators and Targets to feel engaged with one another; Regulators should believe that their recommendations have the potential to affect a Target’s emotional state, and Targets should feel they are implementing recommendations from a real Regulator (Redcay & Schilbach, 2019; Shamay-Tsoory et al., 2019). To capture such interpersonal contingencies, the present study measured brain activity of Regulators and Targets simultaneously with functional magnetic resonance imaging (fMRI) whilst they performed the interpersonal Emotion Regulation Task (iERT; Levy-Gigi & Shamay-Tsoory, 2017). Across trials of this task, a Target viewed negatively valenced images and were asked to down-regulate their emotional reactions by implementing an ER strategy that they had self-selected or had been selected for them by an affiliate Regulator (intra- and inter-personal ER, respectively). Measuring brain activity and behaviours of both Regulators and Targets during the iERT, therefore, allowed us to capture the brain responses of Regulators that drive their selection of ER strategies to recommend, and how the resultant recommendations influence brain responses in Targets during their implementation of Regulator-recommended ER strategies—the unidirectional component of interpersonal ER. This second-person paradigm differs from the “spectator science” that has dominated ER research historically (see Schilbach et al., 2013), whereby brain activity is measured from Regulators whilst they select ER strategies to recommend for anonymous/fictional characters, or from Targets whilst they implement ER strategies recommended by pre-programmed algorithms or representations of agents (e.g., names). Furthermore, to capture as closely as possible the inter-personal ER dynamic as it occurs naturalistically, wherein flexible and situation-sensitive recommendations are more efficacious (Levy-Gigi et al., 2016; Paul et al., 2023), both Regulators and Targets were free to choose between two ER strategies on each iERT trial: the Target could choose or be recommended to disengage attentionally from the image or reappraise it with less negative meaning. We focused on these two ER strategies because they are the most extensively studied (e.g., McRae et al., 2010; Naragon-Gainey et al., 2017). Although both antecedent-focused strategies aim to disrupt the emotion-generative process, they do so at different stages; disengagement reduces early attention to an emotion-evoking situation, whilst reappraisal operates after emotional information has been attended to. Giving both Regulators and Targets a choice between these two ER strategies diverges from the constraints imposed in previous studies, in which Regulators follow predefined instructions (Guendelman et al., 2022; Zhang, Qiu, Tang, & Sun, 2023).

Previous research has identified several factors that moderate the efficacy of inter-personal ER and might exert such effects by influencing neurocognitive mechanisms supporting the process. One such factor is the social proximity between interactants: Targets’ subjective emotional states appear to be regulated more effectively during inter- compared with intra-personal ER when performed between individuals in close relationships, such as friends (Liu et al., 2021; Morawetz et al., 2021; Sahi et al., 2023), romantic couples (Levy-Gigi & Shamay-Tsoory, 2017; Zhang, Qiu, Tang, & Li, 2023), or parent–child dyads (Reindl et al., 2018), but this advantageous effect diminishes or even reverses among newly acquainted participants or strangers (Hallam et al., 2014; Morawetz et al., 2021; Reindl et al., 2018; Zhang, Qiu, Tang, & Sun, 2023). Interestingly, however, some studies have shown that both neural and physiological indices of Targets’ emotional response are regulated more during inter- relative to intra-personal ER even among strangers, despite contradictory subjective experiences (Coan et al., 2006; Ngombe et al., 2022). Another influencing factor is the personality characteristics of the Regulator and Target; studies suggest that autistic traits (Samson et al., 2015), levels of empathic awareness (Guendelman et al., 2022; Nozaki & Mikolajczak, 2020), and proficiency in regulating one’s own emotions quickly and flexibly when faced with negative events (Koole & Fockenberg, 2011) could all contribute to inter-personal ER. For example, Regulators who empathise accurately with a Target’s emotional response, rather than misinterpreting or overly sharing it, should be more likely to recommend emotion regulation strategies that effectively regulate that response. Likewise, Targets with higher proficiency in regulating their own emotions should be more effective in implementing ER strategies recommended to them by Regulators. It is necessary, therefore, to consider such personality characteristics when investigating both stages of inter-personal ER. It is necessary, therefore, to consider such personality characteristics when investigating both stages of inter-personal ER.

To date, no study has investigated patterns of functional brain connectivity that support both Regulators’ selection of ER strategies to recommend *and* their subsequent implementation by Targets. In contrast, network-level analyses of brain imaging data have provided valuable insights into the neurophysiological mechanisms that underpin intra-personal ER; self-directed ER has been shown to involve a modulation of excitatory signals from the dorso- to ventro-lateral prefrontal cortex and inhibitory signals running in the reverse direction (Morawetz et al., 2016). It is proposed that the former reflects an ongoing semantic reinterpretation of emotion-eliciting stimuli, whilst the latter indicates a completion of this process—the final selection of an optimal form of reappraisal. It remains to be seen, however, whether a similar pattern of brain connectivity supports Regulator’s ER strategy selections and/or their subsequent implementation by Targets during inter-personal ER. To investigate this directly, we applied behavioural Dynamic Causal Modelling (bDCM; Rigoux & Daunizeau, 2015) to fMRI data acquired from Regulators and Targets during the iERT. This technique models the directional propagation of neural signals throughout a brain network in response to an external (e.g., visual) stimulus, and how that pattern of effective connectivity is modulated by stimulus characteristics or internal (e.g., cognitive) processes. As an extension of conventional DCM, bDCM then attempts to estimate a behavioural output (e.g., choice) from the pattern of modulated connectivity (Rigoux & Daunizeau, 2015). Importantly, bDCM has not yet been utilised in ER research and studies that have employed conventional DCM have focused predominantly on top–down processes indexed by prefrontal–subcortical connections during the intra-personal implementation of reappraisal (He et al., 2023; Steward et al., 2021; Zhang et al., 2018). To advance this literature, we applied bDCM to identify a pattern of effective connectivity in Regulators that drives their selection of an ER strategy to recommend, determine how these recommendations modulate effective connectivity in Targets during their implementation of Regulator-selected ER strategies, and how this drives the down-regulation of Targets’ subjective emotional responses. To measure the neurocognitive mechanisms supporting these stages of inter-personal ER without any confounding influences associated with social proximity (e.g., relationship type, duration, and level of closeness), we performed fMRI on Regulator and Target dyads who were previously unfamiliar with one another. We then examined whether and how the aforementioned personality characteristics are related to the neurocognitive processes supporting each stage of inter-personal ER.

Driven by Reeck et al.’s (2016) theoretical model, together with evidence of functional brain connectivity patterns during intra-personal ER (Morawetz et al., 2016) and overlapping brain responses in Targets and Regulators during inter-personal ER (e.g., Guendelman et al., 2022; Morawetz et al., 2021), we hypothesised the following: In Regulators, choosing between ER strategies to recommend will elicit directional connectivity from the temporo-parietal cortex (social cognitive system) to the lateral prefrontal cortex (cognitive control system), and their final choice of ER strategy would be associated with modulations of these effective connections. In Targets, we predicted that interpreting Regulator-selected strategies would be associated with excitatory connections from the temporo-parietal cortex to the prefrontal cortex and their implementation would modulate bidirectional connections between the dorsal and ventral prefrontal cortex. Reeck et al.’s (2016) model further proposes that the implementation of Regulator-selected ER strategies should elicit inhibitory signals from prefrontal cortices to the amygdala (emotion-generative system), but we did not evaluate this hypothesis in light of inconsistent meta-analytic data concerning amygdala modulation during intra-personal ER (Buhle et al., 2014; Morawetz et al., 2017). Using bDCM, we then hypothesised that Regulators’ strategy selections and the effectiveness of their recommendations in down-regulating Targets’ subjective emotion responses would be estimated accurately from these patterns of modulated effective connectivity. Finally, in light of theoretical propositions and preliminary research that suggest Regulators’ empathic tendencies (Franklin-Gillette & Shamay-Tsoory, 2021; Guendelman et al., 2022; Nozaki & Mikolajczak, 2020), Targets’ emotion regulation style, and autistic traits might influence the process of inter-personal ER (Koole & Fockenberg, 2011), we explored the following associations: (a) between Regulators’ empathic tendencies, their accuracy in predicting Targets’ upcoming emotional responses, and the effectiveness of Regulator-selected ER strategies at down-regulating those responses; (b) between Targets’ emotion regulation style and autistic traits, and their ability to implement self-selected and Regulator-selected ER strategies; and (c) between these personality characteristics, behaviours on the iERT, and effective connectivity among the social and cognitive control systems.

## 2 Methods

### 2.1 Participants

Fifty-four right-handed Masaryk University students (26 males) without a history of neurological, neurodevelopmental (e.g., autism), or psychiatric diagnoses, and normal or corrected-to-normal vision were recruited for this study. These individuals were paired into 27 age-, sex-, and education-matched dyads. Pair members were previously unfamiliar and introduced to each other for the first time upon their arrival at the Central European Institute of Technology (CEITEC), Masaryk University. Four pairs were removed from the analyses described below due to technical difficulties or a misunderstanding of task instructions by one of the pair members. This resulted in a final sample of 23 pairs (13 male–male), each comprising a Regulator (mean age = 24.7 years; SD = 2.5) and Target (mean age = 24.7 years; SD = 2.5). The majority of this final sample reported to be studying social (43%) and natural (33%) science disciplines. The study was approved by the Ethics Board of the Institute of Psychology, Academy of Sciences in the Czech Republic. All individuals provided written informed consent prior to their participation in the study and were recompensed with 500 Kč (approx. €20.6).

### 2.2 Procedure

Prior to scanning, the members of a given pair were introduced briefly before receiving instructions about the experimental procedure (see Supplementary Materials). They were then assigned randomly to the role of Regulator and Target and this role assignment remained fixed for the duration of the testing procedure. After completing practice trials (see below), each member of the pair was sent to one of two identical scanners housed at CEITEC in which they both underwent synchronised fMRI whilst performing the iERT. After scanning, each member of a given pair completed a set of self-report questionnaires.

### 2.3 Materials

#### 2.3.1 Inter-personal emotion regulation task

**Fig. 1.**
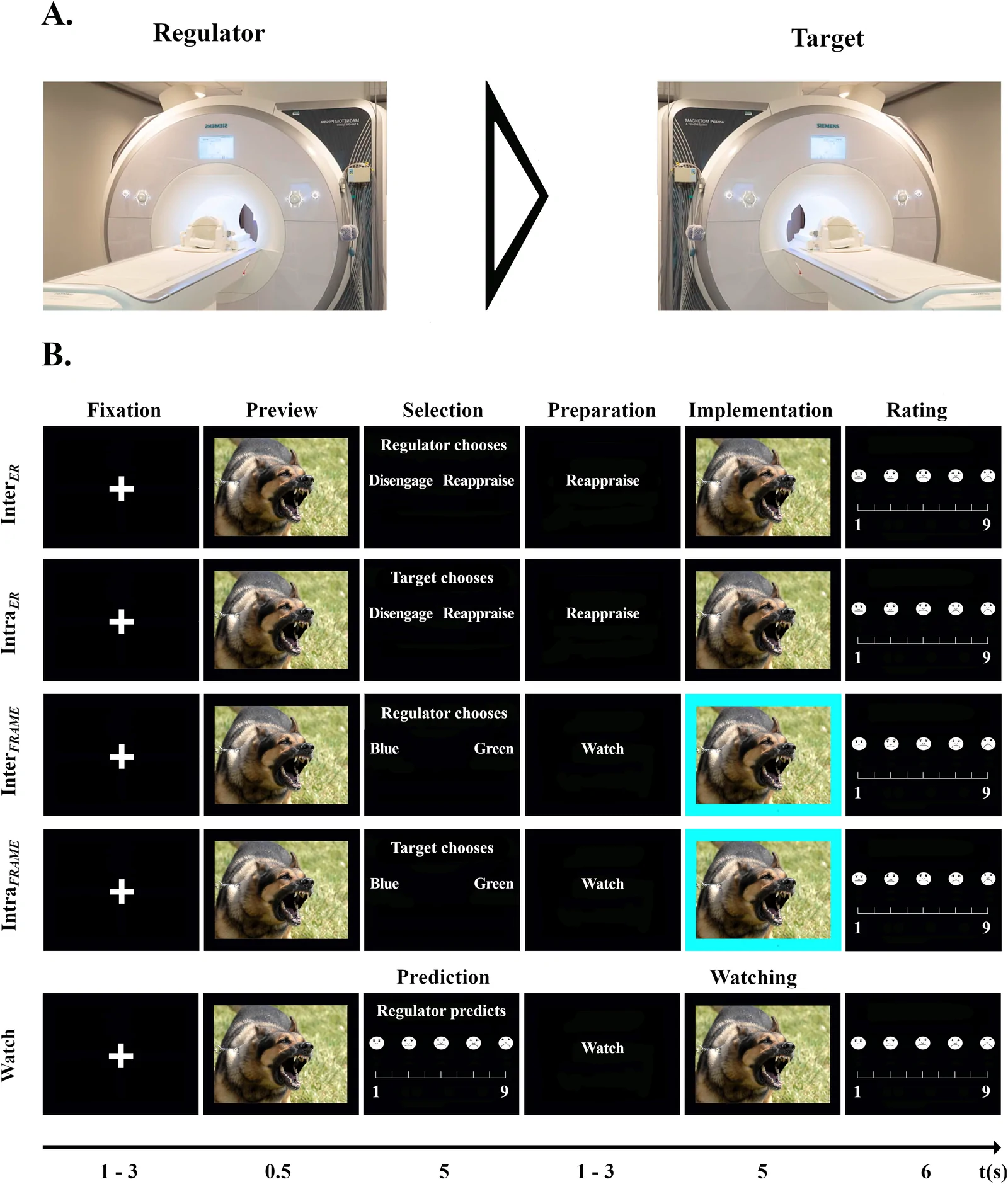
Schematic representation of the five conditions comprising the inter-personal Emotion Regulation Task (iERT). *Note.* Panel (A) is a representation of the physical setup, whereby the Regulator and Target of a given pair were scanned simultaneously in two separate but identical scanners located in the same facility whilst they performed the inter-personal Emotion Regulation Task (iERT) together. A single computer presented stimuli to both participants. Panel (B) is a schematic of trials comprising the five iERT conditions. Inter*_ER_* = Targets implemented Regulator-selected ER strategies; Intra*_ER_* = Targets implemented self-selected ER strategies; Inter*_Frame_* = Targets viewed images surrounded by a Regulator-selected coloured frame without implementing ER; Intra*_Frame_* = Targets viewed images surrounded by a self-selected coloured frame without implementing ER; Watch = Targets passively viewed images without any frame and without implementing ER. Note that the responses of Regulators and Targets were not shown in the Prediction and Rating screens, respectively.

Each Regulator–Target pair performed the Inter-personal Emotion Regulation Task (iERT) whilst undergoing fMRI. This task lasted ~52 minutes and comprised 150 trials—30 for each of 5 conditions (see below), presented in a fully randomised event-related manner. Figure 1 illustrates trials of each condition. All trials were presented via a single presentation computer; thus, both the Regulator and Target of a given pair saw the exact same sequence and timing of stimuli from their respective scanner. The iERT was coded using the Cogent 2000 toolbox (v1.33; https://github.com/lnnrtwttkhn/Cogent2000/).

In the Inter-personal ER condition (Inter*_ER_*), each trial started with a fixation cross of jittered duration, followed by a brief preview of a negatively valenced image. The Regulator was then given a fixed period of 5 sec to select one of two ER strategies—disengagement or reappraisal—for the Target to implement on a subsequent extended presentation of the image to down-regulate their emotional response. The Regulator’s selection was then presented for a jittered duration to allow the Target to prepare the strategy. The previewed image then reappeared and was presented for 5 sec. During this extended presentation, Targets were instructed to try to make themselves “feel less unpleasant” when looking at the image by implementing the Regulator-selected ER strategy; for disengagement they were advised to “think of something completely unrelated” to the content of the image, and for reappraisal they were advised to “change the meaning” of the image. The task instructions presented to Regulators and Targets prior to the experimental procedure provided them with examples of both ER strategies to help guide their selections and implementation (see Supplementary Materials). Finally, Targets were required to rate the intensity of their emotional response to the image on a scale from 1 (*I do not feel unpleasant at all*) to 9 (*I feel very unpleasant*).

Trials of the Intra-personal ER condition (Intra*_ER_*) followed the same sequence of events, but Targets were asked to self-select disengagement or reappraisal and then implement their selected strategy during the extended image presentation. On these trials, Regulators passively waited for Targets to make their self-selection.

In trials of the Inter-personal Frame condition (Inter_Frame_), different instructions were given to Regulators and Targets. After the initial image preview, the Regulator was asked to select between a blue or green frame that would be placed around the image during its extended presentation. Subsequently, Targets were instructed to passively watch the framed image without implementing any ER strategy. Likewise, in the Intra-personal Frame condition (Intra_Frame_), Targets were asked to self-select between these frame colours that would surround the image during its extended presentation and then to passively watch the framed image. By requiring a two-alternative choice similar to that made in the Inter_ER_ and Intra_ER_ conditions, the Frame trials controlled for domain-general/ER-unspecific decision-making and perceptual-motor processes involved in action selection. The structure of Frame trials also maintained the same inter- and intra-personal context in which choices were made, controlling for ER-unspecific agency processing; Regulators chose colours for Targets and Targets were then faced with Regulators’ choices, or Targets chose colours for themselves and were then faced with their own choices. This allowed us to isolate neural processes specific to ER strategy selection and implementation in intra- and inter-personal contexts, and evaluate the independent effectiveness of inter- and intra-personal ER. Importantly, participants were entirely unconstrained in their choices of ER strategies and frame colours.

Finally, we also included a Watch condition. On these trials, Regulators were asked to predict the Targets’ eventual intensity rating for each previewed picture prior to its extended presentation and Targets were instructed to rate their emotional response to the unframed image without implementing ER. These trials were not considered in fMRI analyses but allowed us to assess whether Regulators’ accuracy in predicting the Target’s emotional state was related to Regulators’ personality characteristics and/or the effectiveness of Regulator-selected strategies on Inter*_ER_* trials.

Both Regulators and Targets selected the disengagement or blue frame option by using their left thumb to press the left-most key of a four-button response box, and selected the reappraise or green frame option by using their right thumb to press the right-most key of the response box. Since Regulators and Targets saw the exact same stimuli and the order of trials was fully randomised, it was possible that Regulators’ predictions on early Watch trials could have influenced Targets’ subsequent ratings during Inter*_ER_* and Intra*_ER_* trials. Likewise, Targets’ ratings on early Inter*_ER_* and/or Intra*_ER_* trials could have influenced Regulators’ predictions on later Watch trials. To avoid this, Regulators made their predictions on Watch trials and Targets provided their ratings on all trial types by using their right thumb to press the right-most key of the response box the number of times corresponding to their prediction or rating, but their final prediction or rating was not shown on the Prediction/Rating screens. To familiarise participants with this process and give Targets an opportunity to practice implementing each of the ER strategies, each Regulator–Target pair practiced 15 trials of the iERT (3 trials per condition) outside of the scanner. The images used in these practice trials were not included in the scanning procedure.

#### 2.3.2 Stimuli

Thirty negative images (e.g., crying infants, mutilations) were selected from the International Affective Picture System (Lang et al., 2005) on the basis of their normative valence and arousal ratings (Supplementary Table S1). Specifically, we chose a set of images with medium to high negative valence (range = 1.5 and 3.9, *Mdn* = 2.6) and moderate to strong arousal (range = 4.0–7.3, *Mdn* = 5.7). This provided us with an equal number of images categorised as “low” (*Mdn* = 5.1) and “high” arousal (*Mdn* = 6.4; Z = - 4.669, *p* < .001, *r* = -0.85). Since negative images with varying arousal yield different preferences for reappraisal and disengagement (Sheppes et al., 2011, 2014), this stimulus set was chosen to maximise variability in Regulators’ and Targets’ ER strategy selections. Each image was presented to participants once in each condition.

### 2.4 Self-report measures

The Autism Spectrum Quotient (AQ; Baron-Cohen et al., 2001) measures participants’ autistic traits. This 50-item questionnaire consists of statements (e.g., “*I find it easy to ‘read between the lines’ when someone is talking to me.*”) that assess different domains (e.g., social skills, attention to detail, communication), to which participants respond on a 4-point Likert scale ranging from 1 (“*Definitely agree*”) to 4 (“*Definitely disagree*”). The AQ showed good internal consistency across the present sample (Cronbach’s α = .80).

The Interpersonal Reactivity Index (IRI; Davis, 1983) measures empathic tendencies through 28 statements mapping onto 4 dimensions of empathy: empathic concern (EC; e.g., “*When I see someone being taken advantage of, I feel kind of protective towards them*.”), personal distress (PD; e.g., “*In emergency situations, I feel apprehensive and ill-at-ease.*”), fantasy (FS; e.g., “*When I watch a good movie, I can very easily put myself in the place of a leading character*.”), and perspective taking (PT; e.g., “*Before criticising somebody, I try to imagine how I would feel if I were in their place*.”). Together, the first two dimensions assess affective empathy—that is, the extent to which participants experience emotions of concern and distress for others. The latter two dimensions assess cognitive empathy—the self-perceived ability to infer the emotional state and perspective of fictional characters and other people. Each item is responded to on a 5-point Likert scale ranging from 0 (“*Does not describe me well*”) to 4 (“*Describes me very well*”). There was acceptable reliability for all dimensions in the present sample (Cronbach’s α ranging from .62 to .81).

The Action Control Scale (ACS; Kuhl & Beckmann, 1994) was used to assess participants’ tendency for action- or state-oriented ER. Action orientation relates to tendencies toward taking decisive actions and initiative, whilst state-orientated ER refers to indecisiveness and rumination (Jostmann & Koole, 2007). The construct of action control, therefore, encompasses self-regulation and cognitive control. The 36-item instrument comprises statements with two possible options that tap into three dimensions of action orientation: failure-related (AOF; e.g., “When I’ve worked for weeks on one project and then everything goes completely wrong: (A) *It takes me a long time to get over it*. (B) *It bothers me for a while, but then I don´t think about it anymore*.”), decision-related (AOD; e.g., “When I have work to do at home: (A) *It is often hard for me to get started*. (B) *I usually get started right away*.”), and performance-related (AOP; e.g., “When I have learned a new and interesting game: (A) *I quickly get tired of it and do something else*. (B) *I can really get into it for a long time*.”). Higher scores represent a greater action- (or goal-) oriented ER tendencies. Acceptable internal consistency was observed within our sample (AOF *α* = .58; AOD *α* = .70, and AOP *α* = .64). As AOF and AOP occasionally load onto the same factor, and AOP is related more closely to positive emotions, herein we focus only on AOF and AOD.

This study was focused specifically on furthering our understanding of inter-personal ER as it unfolds dynamically between Regulators and Targets in naturalistic settings. Since measures that assess habitual ER (e.g., the Interpersonal Emotion Regulation Questionnaire; Hofmann et al., 2016) seem to be less capable of capturing the context-dependent choices made in specific interactions (Koval et al., 2023), we did not use them in the present study.

### 2.5 MRI data acquisition

Structural and functional images from both members of a given dyad were acquired simultaneously using 2 identical 3T Siemens Prisma scanners, with 64-channel bird-cage head coils. A high-resolution T1-weighted anatomical image was first acquired (TR = 2300 ms, TE = 2.33 ms, flip angle = 8°, FoV = 224 mm, 1 mm^3^ isotropic voxels), followed by a functional whole-brain time-series obtained with a T2*-weighted EPI sequence (34 axial slices, slice thickness = 4 mm, interleaved slice order; TR = 2000 ms, TE = 35 ms, flip angle = 60°, FoV = 204, 3 x 3 x 4 mm^3^ voxels). Functional time-series were acquired from each member of a pair in a single functional run comprising 1570 volumes. An external programmable signal generator (www.siglent.com) initiated synchronous acquisition sequences in both scanners.

### 2.6 MRI data pre-processing

Structural and functional brain images were preprocessed using various functions packaged within FMRIB’s software library (FSL; Jenkinson et al., 2012). First, anatomical images were skull stripped using BET. Then, functional time-series were preprocessed with FEAT: volumes were slice-time corrected, a 100-sec temporal high-pass filter was applied to counteract scanner drift, images were spatially smoothed using a Gaussian Kernel with FWHM of 5 mm, and motion correction was performed with MCFLIRT. Using FLIRT (Jenkinson et al., 2002), the preprocessed time-series were then registered to participants’ brain-extracted structural image using a linear Boundary-Based Registration transformation, and subsequently to the MNI152 brain using a linear transformation with 12 DOFs.

To detect and remove any artifactual signals that remained after preprocessing, we performed independent component analysis on the time-series with MELODIC (Beckmann & Smith, 2004) to identify 50 primary component signals that were categorised automatically by the Spatially Organised Component Klassifikator (Bhaganagarapu et al., 2013). Artifactual components reflecting residual motion or physiological noise were regressed out of participants’ time-series using *fsl_regfilt* before any first- or higher-level analyses were run.

### 2.7 Analyses

#### 2.7.1 Behavioural data

**Fig. 2.**
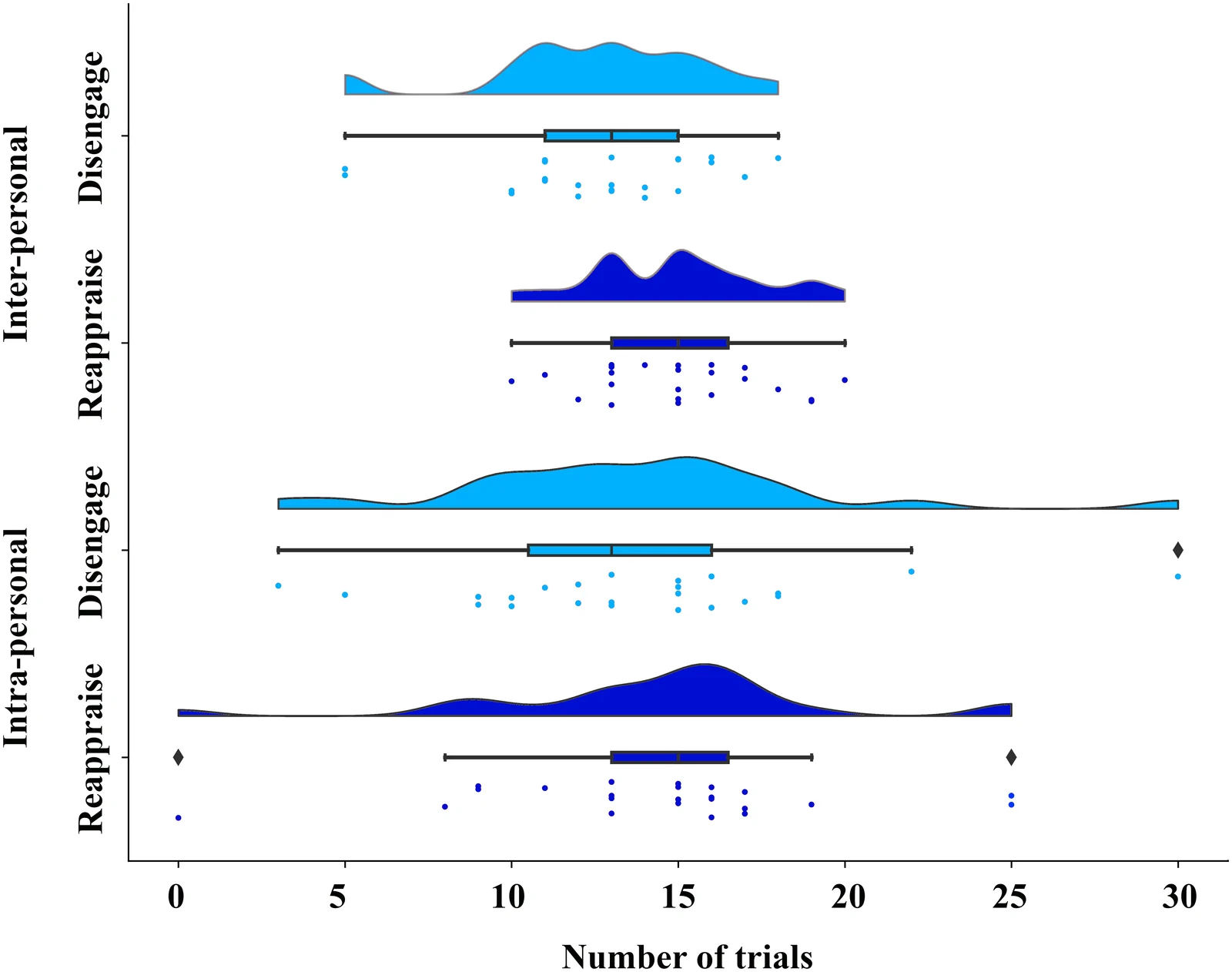
Distributions of Disengage and Reappraise strategy selections on Inter- and Intra-personal ER trials. *Note.* Plots present the number of trials in which Disengage (*light blue*) and Reappraise (*dark blue*) were selected by Regulators during inter-personal ER (*top*) and by Targets during intra-personal ER (*bottom*). Boxplots present the medians and interquartile ranges. Extreme values (±3 SD from the mean) are highlighted as triangles within the dot plots.

To evaluate the effectiveness of inter-personal ER in down-regulating Targets’ subjective emotional reactions to the negative images, we compared their intensity ratings after the implementation of reappraisal or disengagement during the Inter*_ER_* and Intra*_ER_* conditions with those from Inter*_Frame_* and Intra*_Frame_* trials. As illustrated in Figure 2, giving both Regulators and Targets a choice of two ER strategies resulted in an unequal number of trials in which each ER strategy was selected. To account for this trial imbalance, Linear Mixed Effects Modelling (LMM) was employed for comparisons of Targets’ ratings. We employed a data-driven step-up approach to determine the optimal model: the main effects of Condition (Inter*_ER_* and Intra*_ER_*), Trial Type (Disengagement, Reappraisal and Frame), and the Condition-by-Trial Type interaction were added sequentially first as fixed and then as random effects, and the change in model fit at each step was evaluated using Akaike’s Information Criteria (Sakamoto et al., 1986).

To examine relationships between personality characteristics, brain responses, and behavioural performance on the iERT, we defined the following behavioural indices: (1) the mean absolute difference between Regulators’ predictions and Targets’ actual intensity ratings on Watch trials, with lower values representing greater prediction accuracy (less deviation from Targets’ actual ratings; *Prediction*); (2) the mean difference between Targets’ intensity ratings on Inter*_Frame_* compared with Inter*_ER_* trials, with higher positive values representing greater effectiveness of Regulator-selected ER strategies in down-regulating Targets’ emotional responses (Efficacy*_Inter_*); and (3) the mean difference between Targets’ intensity ratings on Intra*_Frame_* and Intra*_ER_* trials, with higher positive values representing greater effectiveness of self-selected ER strategies (Efficacy*_Intra_*).

#### 2.7.2 fMRI data: General linear modelling

We performed general linear modelling (GLM) to localise brain responses during Regulators’ selections and Targets’ implementation of Regulator- and self-selected ER strategies. At the participant level, fixed-effects analyses were performed with FEAT to capture blood-oxygen level-dependent (BOLD) responses associated with each of the six sub-trial events comprising Inter*_ER_*, Intra*_ER_*, Inter*_Frame_*, and Intra*_Frame_* trials: Fixation, Preview, Selection, Preparation, Implementation, and Rating (see Fig. 1B). These events were convolved with a double-gamma haemodynamic response function, and pre-whitening was applied with FILM to combat temporal autocorrelations (Woolrich et al., 2001).

In line with our aims, we then performed the following contrasts between specific sub-trial events. First, we contrasted BOLD responses during the 5-sec Selection events of Inter*_ER_* trials (i.e., when Regulators selected an ER strategy for Targets) with those evoked during the same events of Inter*_Frame_* trials (i.e., when Regulators selected a frame colour for Targets; Selection Inter*_ER_* > Inter*_Frame_*). Second, to isolate brain responses associated specifically with Targets’ ER strategy selections from those associated with their self-selections, we contrasted BOLD responses during the Selection events of Intra*_ER_* trials with those during the same events of Intra*_FRAME_* trials (i.e., when Targets self-selected a strategy; Selection Intra*_ER_* vs. Intra*_FRAME_*). Third, we contrasted BOLD responses during the 5-sec Implementation events of Inter*_ER_* trials (i.e., when Targets implemented Regulator-selected ER strategies) with those during the same events of Inter*_Frame_* trials (i.e., when Targets viewed images surrounded by Regulator-selected frames; Implementation Inter*_ER_* vs. Inter*_Frame_*). Fourth, to isolate brain responses associated specifically with Targets’ implementation of Regulator-selected ER strategies from those associated with their implementation of self-selected ER strategies, we contrasted the Implementation events of Intra*_ER_* trials with the same events of Intra*_FRAME_* trials (i.e., when Targets implemented self-selected ER strategies; Implementation Inter*_ER_* vs. Intra*_ER_*). For each contrast, the remaining sub-trial events were modelled as regressors of no interest.

Contrast estimates from these first-level analyses were carried forward for group-level analyses, where they were evaluated with non-parametric permutation inference to achieve more precise control over false positives than other cluster-based methods (Eklund et al., 2016). Specifically, whole-brain *t*-maps emerging from each contrast underwent 5000 permutations with threshold-free cluster enhancement with the *randomise* function. This process produced whole-brain *p*-value maps for each contrast that reflect the significance of *t*-values expressed at each voxel; that is, the significance with which each voxel expresses a given contrast. These *p*-value maps were thresholded at *p* < .05 to localise clusters of significant activation emerging from each contrast.

#### 2.7.3 fMRI data: Behavioural dynamic causal modelling

As detailed above, bDCM describes the mathematical approach of (i) modelling the directional propagation of neural signals throughout a brain network in response to an external stimulus, (ii) how that pattern of effective connectivity is modulated by stimulus characteristics or cognitive processes, and (iii) attempts to estimate a behavioural output (e.g., choice) from the pattern of modulated connectivity (Rigoux & Daunizeau, 2015). Using the Variational Bayesian Analysis toolbox of MATLAB (Daunizeau et al., 2014), we applied separate bDCMs to the fMRI data of Regulators and Targets to evaluate inferences of model parameters rather than model structure (Stephan et al., 2010): For Regulators, we investigated whether a pre-defined pattern of effective connectivity was modulated during their selection of ER strategies on Inter*_ER_* trials, and whether their eventual selection on these trials could be estimated from that modulated connectivity pattern. For Targets, we assessed whether the same pattern of effective connectivity was modulated during their subsequent implementation of the Regulator-selected ER strategy on Inter*_ER_* trials, and whether that modulated pattern could be used to estimate whether or not the Target’s rating for a given image on these trials was down-regulated relative to the corresponding Inter*_Frame_* trial.

Each bDCM comprised three parameter matrices: Matrix A characterised directional endogenous functional connections among five left-lateralised nodes. For both Regulators and Targets, four of these nodes were modelled as spheres of 5 mm radius centred on coordinates reported in Morawetz et al. (2021). These brain regions are implicated consistently in the cognitive control of emotions, as shown in the meta-analysis by Morawetz et al. (2017). Importantly, these spheres encompassed overlapping localisations from our GLM analyses (see Fig. 4). A fifth input node was added in the primary visual cortex. From each node, we extracted the first eigenvariate of BOLD time-series from all voxels in participants’ native space that expressed a contrast of interest in the GLM analyses (F-value at *p* < .05, uncorrected). For Regulators, this was the Inter*_ER_* > Inter*_Frame_* contrast applied to the Selection events; for Targets, this was the Inter*_ER_* > Inter*_Frame_* contrast applied to the Implementation events. One Regulator was excluded from the subsequent analysis because no voxels expressed this contrast at the threshold level in one of the nodes. Effective connections among these five nodes were then modelled according to the findings of Morawetz et al. (2016)—a study that empirically determined the best fitting of 18 models comprising intrinsic connections among the same nodes and their modulation during intra-personal ER implementation. For both Regulators and Targets, an input node was located in the primary visual cortex (V1; x = -8, y = -86, z = 2) using the central coordinates of V1 according to the WFU Pick Atlas. From here, signals were projected unidirectionally onto the supramarginal gyrus (SMG; x = -51, y = -56, z = 30) and subsequently to the middle frontal gyrus (MFG; x = -43, y = 13, z = 42). From the MFG, bidirectional endogenous connections then permitted the propagation of neural signals to the supplementary motor area (SMA; x = -2, y = 17, z = 53) and inferior frontal gyrus (IFG; x = −48, y = 21, z = −1).

Matrix B introduced task-dependent (trial-by-trial) modulations of the endogenous connections from SMG to MFG and between MFG and IFG. For Regulators, a single modulatory input was modelled as the unconvolved onsets of Selection events during Inter*_ER_* trials. For Targets, two modulators were modelled that comprised the onsets of Implementation events during Inter*_ER_* trials in which Regulators selected Reappraise or Disengage. In this way, the endogenous network of Targets could be modulated by the trial-specific ER strategy selected by the Regulator. Matrix C modelled the influence of visual stimuli on the input V1 node during the onset of Selection (Regulators) and Implementation events (Targets) during all Inter*_ER_*, Intra*_ER_*, Inter*_Frame_*, or Intra*_Frame_* trials.

## 3 Results

### 3.1 Behavioural data

**Fig. 3.**
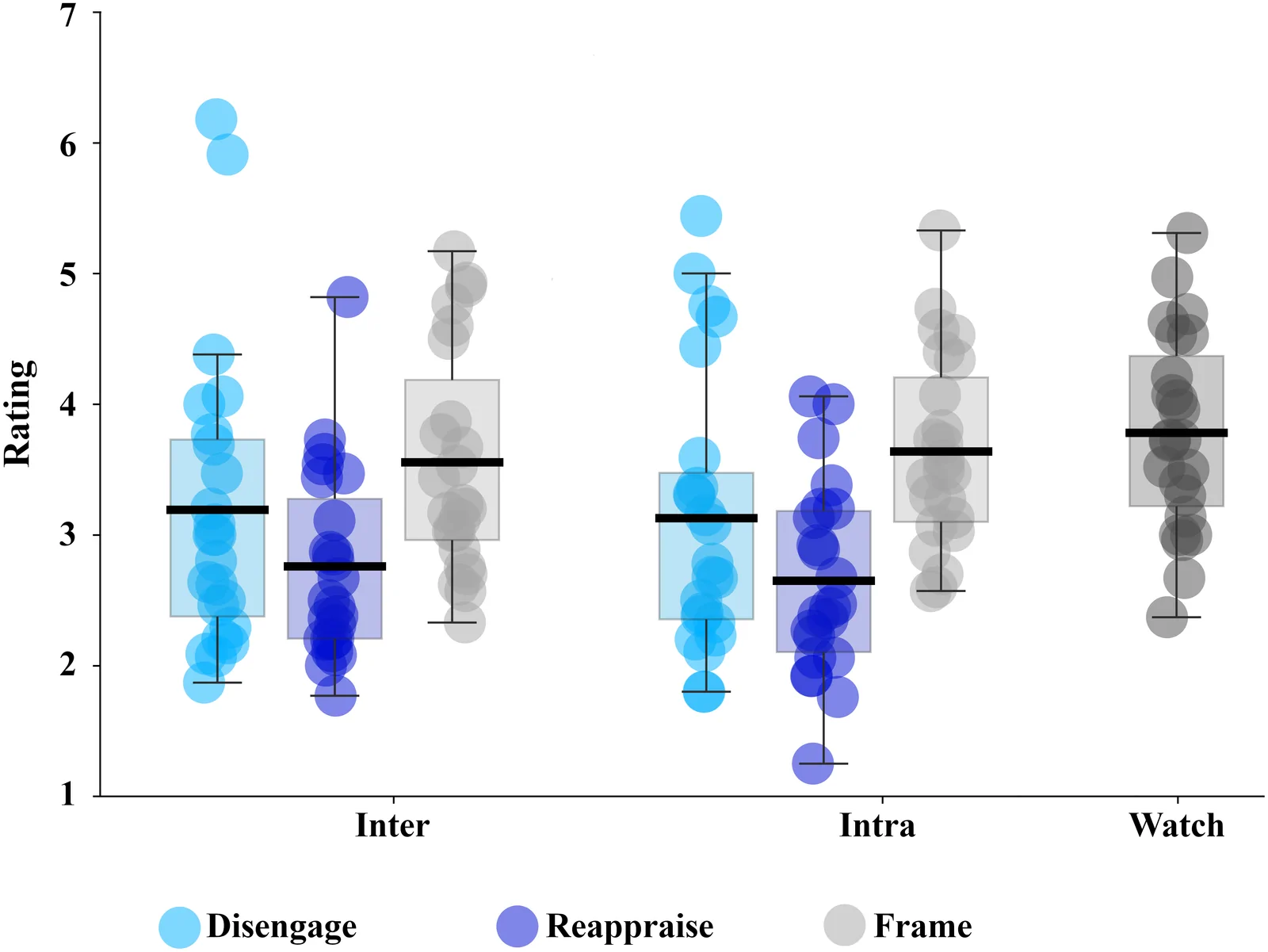
Target intensity ratings across trial types. *Note.* Inter/Intra = Inter-/Intra-personal condition; Targets’ intensity ratings in Watch condition are presented separately, because they were not influenced by the Regulator.

The distributions of Targets’ intensity ratings across the iERT conditions are illustrated in Figure 3. To these data we applied the optimal LMM emerging from our step-up approach (see Supplementary Table S2). This model contained the fixed effects of Condition with the levels Inter- and Intra-personal, Trial Type with the levels Frames, Disengagement, and Reappraisal, and the Condition-by-Trial Type interaction; and a random intercept and Trial Type effect. A formal specification of the model is given below:

$$Rating_{ij}\;=\;\beta_{0}\;+\;u_{0j}\;+\;\beta_{1}{\left(Condition\right)}_{i}\;+\;\beta_{2}{\left(Trial\;Type\right)}_{i}\;+\;\beta_{3}{\left(Condition\;x\;Trial\;Type\right)}_{i}\;+\;u_{1}{\left(Trial\;Type\right)}_{ij}\;+\;\varepsilon_{ij}$$ (1)

In equation (1), fixed effects (β) represent population-level relationships that remain consistent across all trials (*i*) and random effects (*u)* capture deviations from these population-level effects for each pair (*j*). Following best practices for multi-level data, Trial Type was included as both a fixed effect to estimate its overall influence across participants and as a random effect to account for individual variability (Barr et al., 2013). Likewise, the fixed intercept (β*_0_*) reflects the overall mean rating whilst the random intercept (*u_0j_*) accounts for pair-specific deviations in baseline ratings.

Table 1 presents the results of this LMM. A significant main effect of Trial Type (*p* < .001) emerged; post-hoc comparisons revealed significantly stronger emotional responses on Frame trials (3.65 [±.16]) relative to both Disengage (3.10 [±.17]; *p* = .003) and Reappraise trials (2.79 [±.17]; *p* < .001), and on Disengage compared with Reappraise trials (*p* = .037). No other significant effects were observed.

**Table 1.** Estimates of fixed and random effects from the linear mixed model.

Fixed effects				
	Estimates/β	95% CI	*t*	*p*
Intercept	3.64	[3.31, 3.97]	21.45	<.001
Condition (inter vs intra)	-0.08	[-0.28, 0.11]	-0.82	.412
Trial type (frame vs disengage)	-0.45	[-0.75, -0.15]	-2.94	.003
Trial type (frame vs reappraise)	-0.99	[-1.27, -0.70]	-6.81	<.001
Condition (inter vs intra) x trial type (frame vs disengage)	0.03	[-0.31, 0.37]	-0.17	.868
Condition (inter vs intra) x trial type (frame vs reappraise)	0.17	[-0.16, 0.51]	1.01	.314
Random effects				
	Estimates	Variance		
Pair (intercept)	0.57	0.55		
Trial type (frame vs disengage)	0.26	0.20		
Trial type (frame vs reappraise)	0.20	0.13		
Model fit				
	Marginal	Conditional		
R^2^	.03	.18		< .001

*Note*. After removing trials with missing ratings (N = 65), analyses were based on 2695 observations from 23 pairs. Reference categories were Intra*_ER_* and Frame for the Condition and Strategy variables, respectively. The fixed effect intercept represents the estimated mean rating across all conditions for all pairs, whilst the random intercept for Pair captures for pair-specific deviations from this mean. Confidence intervals for the fixed effects were computed using the Wald method within the *confint.merMod()* function of the lme4 package. Model fit was estimated following Johnson’s (Johnson, 2014) approach, incorporating both fixed and random effects. Estimates of fixed effects represent the expected change in ratings per unit change in each predictor averaged across the entire sample. In contrast, random effects estimates (Best Linear Unbiased Predictors) indicate the average magnitude of pair-specific deviations, regardless of direction, whilst random effect variances describe their overall spread.

Earlier studies have reported that reappraisal is preferred over disengagement in response to low-arousal negative images, but the reverse is true for high-arousal stimuli (e.g., Sheppes et al., 2014). Although this is beyond the scope of the current study, we performed supplementary analyses to assess whether such preferences were shown during inter- and intra-personal ER on the iERT and how this affected Targets’ ratings. The results of these analyses are presented in Supplementary Tables S3–S5.

### 3.2 fMRI data: General linear modelling

In Regulators, a diffuse but predominantly left-lateralised pattern of brain response was engaged more strongly whilst they selected an ER strategy compared with when they selected a frame colour for Targets (Selection Inter*_ER_* > Inter*_Frame_*)*.* This pattern spanned the frontal pole, dorso-lateral and medial prefrontal cortex (e.g., supplementary motor area), posterior cingulate and precuneus, lateral temporal and temporo-parietal cortex, and the right cerebellum. In Targets, a similarly diffuse and left-lateralised pattern of brain responses was engaged more strongly during their self-selection of ER strategies relative to frame colours (Selection Intra*_ER_* > Intra_F_*_rame_*) comprising dorso-lateral and medial prefrontal cortex, and temporo-parietal cortex. These patterns appear to differ between Regulators and Targets in the involvement of the left ventro-lateral prefrontal cortex and subcortical nuclei (pallidum and caudate nucleus), but the between-subject contrast [Selection Inter*_ER_* > Inter*_Frame_*] > [Selection Intra*_ER_* > Intra*_Frame_*] revealed no voxels expressing this comparison significantly (*p*_corr_ > .065).

**Fig. 4.**
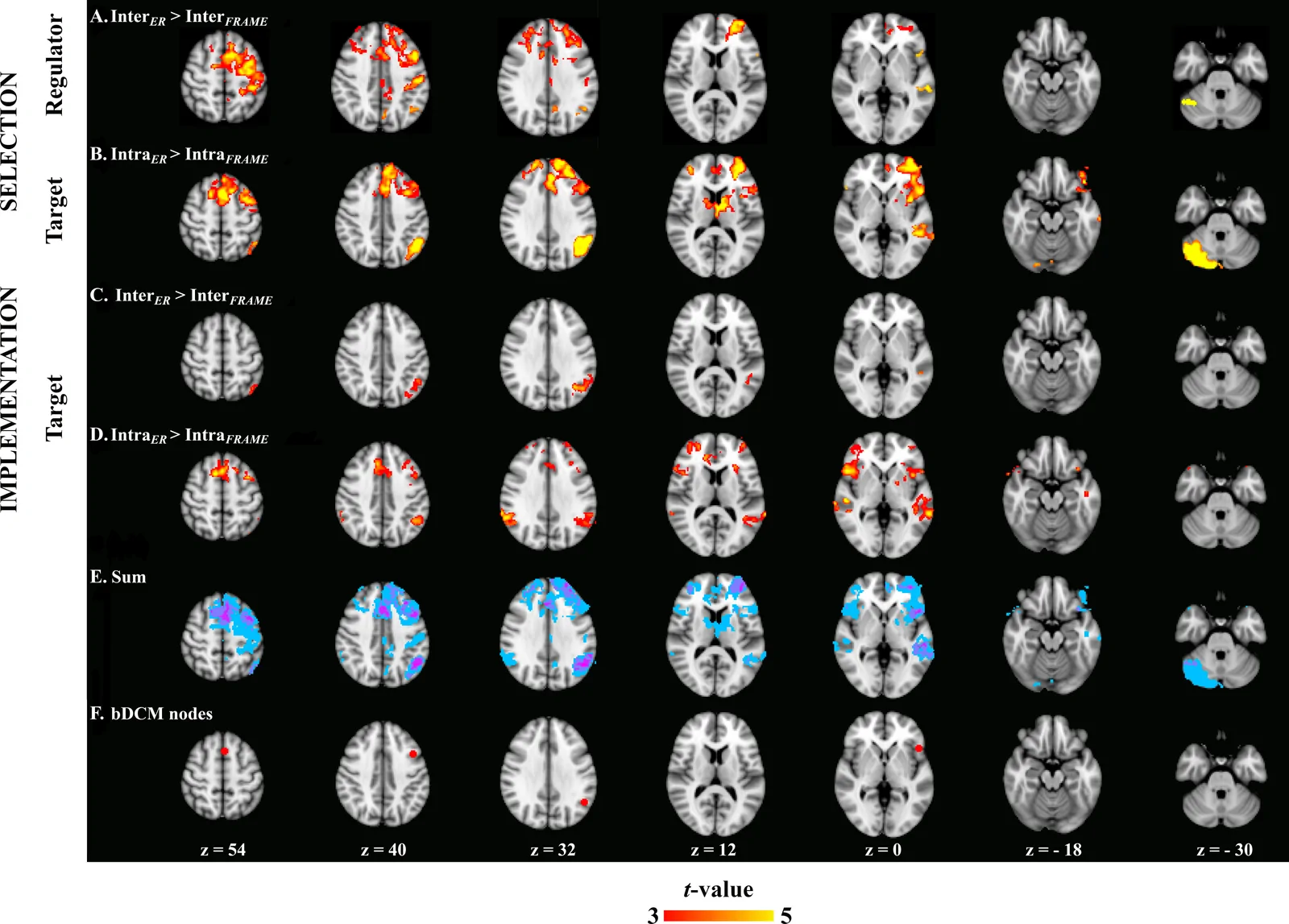
Localisations from GLM results. *Note.* Row (A–D): the results of group-level contrasts for which significant clusters emerged after non-parametric permutations (*p*_corr_ < .050). Resulting *t*-maps are superimposed over the MNI template, thresholded at *t* > 3.0. Row (E) shows the sum of the binarised thresholded *p*-maps (*p*_corr_ > .050) from each of the four contrasts presented above; this image ranges from 1 (*light blue*) to 4 (*bright purple*), indicating the number of intersections (overlapping localisations). A value of 4, therefore, corresponds to the conjunction of these contrasts. Row (F) shows four of the five nodes (spheres of 5 mm radius) from which BOLD responses were extracted for the bDCM. These nodes were centred on coordinates reported by Morawetz et al. (2017) that correspond with intersections in row (E) (values >3); a fifth node (V1; not illustrated) was added later. Brain images and GLM results are presented in radiological orientation (left=right).

In Targets, stronger brain responses were observed during their implementation of Regulator-selected ER strategies compared with when they viewed images surrounded by Regulator-selected coloured frames (Implementation Inter*_ER_* > Inter*_Frame_*) within a single left-hemisphere cluster encompassing the temporo-parietal and lateral temporal cortex (middle temporal gyrus). Stronger brain responses in Targets’ whilst they implemented self-selected ER strategies compared with viewing images surrounded by self-selected coloured frames (Implementation Intra*_ER_* > Intra*_Frame_*) comprised a far more diffuse and bilateral pattern of responses, spanning the medial prefrontal (e.g., supplementary motor area), dorso-lateral and ventro-lateral prefrontal cortices, frontal poles, anterior insula, lateral temporal, and temporo-parietal cortices. However, no voxels expressed the within-subject contrast implementation Inter*_ER_* *>* Intra*_ER_* significantly (*p*_corr_ > .137). These GLM results are presented in Figure 4 and Supplementary Table S6.

Since brain responses habituate to repeated exposures of emotion-inducing stimuli, it is unsurprising that both Regulators and Targets showed habituation across the five successive presentations of images—once in each condition (see Supplementary Fig. S3). Importantly, however, because we fully randomised the order in which the five conditions were presented to participants, these habituation effects were distributed evenly across the conditions and could not exert systematic effects on the aforementioned contrasts of brain responses or comparisons of ratings between conditions.

Summing the thresholded parametric maps emerging from the contrasts illustrates strong overlap within the left SMA, MFG, IFG, and SMG during ER strategy selections made by Regulators (Selection Inter*_ER_* > Inter*_Frame_*) and Targets (Selection Intra*_ER_* > Intra*_Frame_*). Moreover, this pattern of overlap comprises many of the left-hemisphere clusters of brain response elicited more in Targets during their implementation of self-selected ER strategies (Implementation Intra*_ER_* > Intra*_Frame_*), and the temporo-parietal cluster engaged during their implementation of Regulator-selected strategies (Implementation Inter*_ER_* > Inter*_Frame_*). In light of this overlap, and because a pattern of effective connectivity among these brain regions during intra-personal ER has been determined previously (Morawetz et al., 2016), we used bDCM to investigate whether this pattern of effective connectivity was modulated in Regulators and Targets during the iERT.

### 3.3 fMRI data: Behavioural dynamic causal modelling

**Fig. 5.**
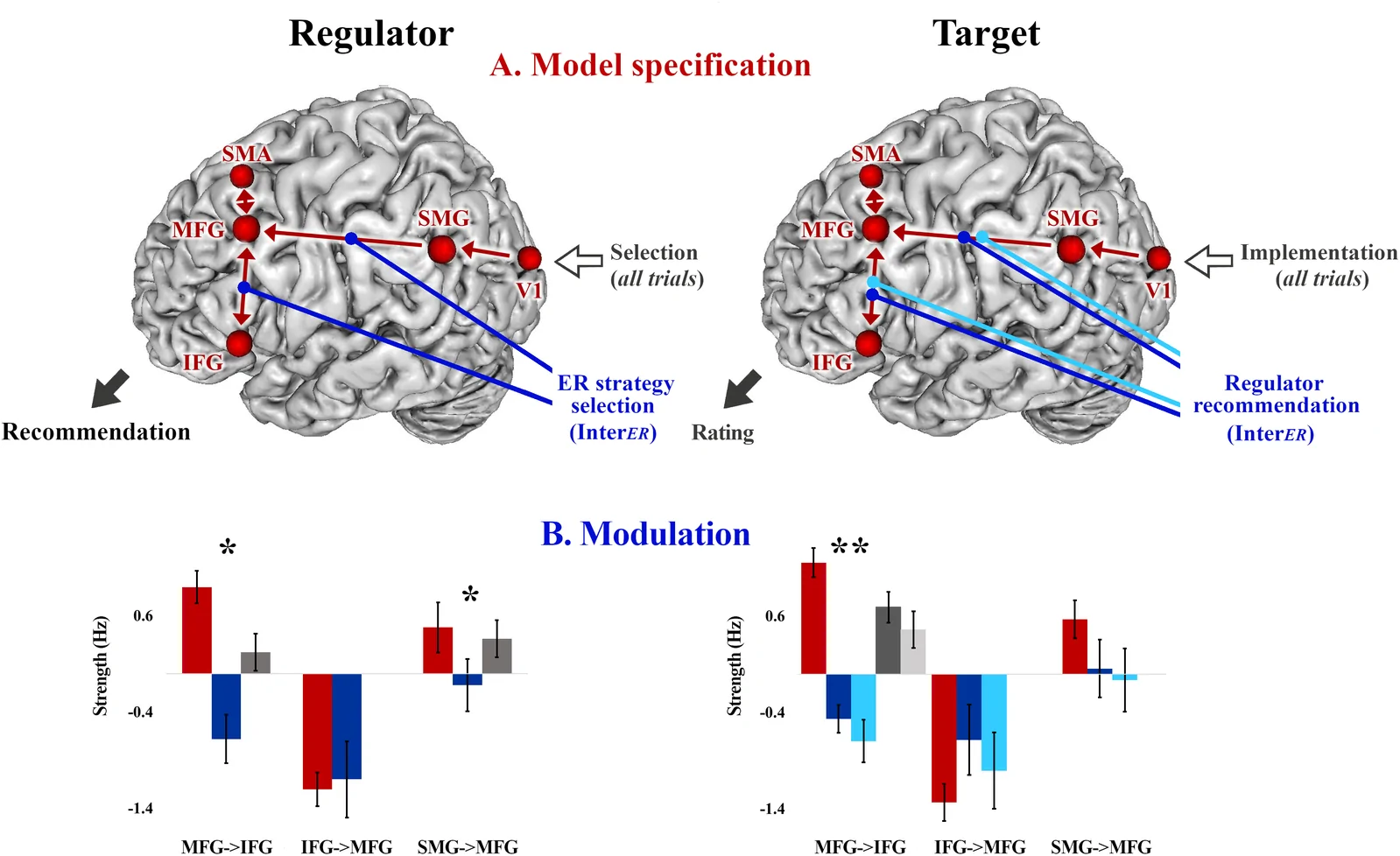
The specification and results of Behavioural DCMs applied to Regulators and Targets. *Note*. Panel (A) illustrates the models comprising the same left-lateralised five-node network applied to brain imaging data acquired from individual Regulators (*left*) and Targets (*right*). Unfilled grey arrows indicate the trial-by-trial visual input that entered the network through V1—the onsets of Selection or Implementation events across Inter*_ER_*, Intra*_ER_*, Inter*_Frame_*, and Intra*_Frame_* trials. Red arrows show the modelled direction of endogenous connectivity between nodes in response to the visual input. Blue arrows identify endogenous connections that could be modulated during specific events of Inter*_ER_* trials: for Regulators, such modulation could occur during the selection of ER strategies to recommend; and for Targets, the modulation of these endogenous connections could occur during the implementation of Regulator-selected reappraisal (*dark blue*) or disengagement (*light blue*). Filled grey arrows indicate the behavioural response that was estimated from these patterns of modulated effective connectivity—either the selection made by Regulators or the change in Targets’ intensity rating for a given image after implementing a Regulator-selected ER strategy on Inter*_ER_* trials relative to viewing the image surrounded by a Regulator-selected coloured frame on the corresponding Inter*_Frame_* trial. In panel (B), red bars plot the strength of endogenous connections, blue bars illustrate the degree to which those connections were modulated during Selection events in Regulators and Implementation events in Targets, and grey bars depict total connectivity for connections that were modulated significantly (*p* < .05). In Targets, modulations and total connectivity during the implementation of reappraisal and disengagement are shown in dark or light blue or grey, respectively. *Abbreviations*: V1 = primary visual cortex, SMG = supramarginal gyrus, SMA = supplementary motor area, MFG = middle frontal gyrus, IFG = inferior frontal gyrus; *p < .050.

Parameters estimated by the bDCM are illustrated in Figure 5 and specified in Table 2. These results show the most likely pattern of endogenous connectivity among the five nodes during Selection events for Regulators and Implementation events for Targets, across all Inter*_ER_*, Intra*_ER_*, Inter*_Frame_*, and Intra*_Frame_* trials. These results also show how the strength of certain endogenous connections is modulated downward in Regulators during their selection of ER strategies and in Targets whilst they implement these Regulator-selected ER strategies on Inter*_ER_* trials. From these modelled patterns of effective connectivity, we correctly estimated Regulators’ ER strategy selections on 74.03% (±2.40) of Inter*_ER_* trials, achieving 67.70% (±5.03) sensitivity and 74.66% (±5.57) specificity. For Targets, the model correctly estimated whether their intensity ratings for images presented on Inter*_Frame_* trials were down-regulated during the implementation of Regulator-selected ER strategies on 89.91% (±2.71) of Inter*_ER_* trials, with 87.51% (±3.12) sensitivity and 90.62% (±3.00) specificity. For all Regulators and Targets, the model evidence for the bDCMs was higher than that of a null (trivial) model (see Supplementary Fig. S1).

**Table 2.** Model parameters for Regulators and Targets.

	Regulators Selection				Targets Implementation			
	M	*t*	*p*	CI	M	*t*	*p*	CI
Endogenous connectivity (Matrix A)								
V1→SMG	.22	1.1	.143	[-.20, .64]	.13	0.75	.230	[-.23, .50]
SMG→MFG	.42	1.84	.040	[-.05, .90]	.50	2.89	.004	[.14, .86]
MFG→SMA	.86	5.72	<.001	[.55, 1.17]	.79	6.2	<.001	[.52, 1.05]
SMA→MFG	.60	2.31	.016	[.06, 1.14]	.56	1.93	.033	[-.04, 1.16]
MFG→IFG	.79	5.33	<.001	[.48, 1.10]	1.02	7.69	<.001	[.74, 1.29]
IFG→MFG	-1.05	-6.86	<.001	[-1.37, -.73]	-1.16	-6.89	<.001	[-1.52, -.81]
Modulation (Matrix B)								
MFG→IFG	-.59	-6.27	<.001	[-1.84, -.92]				
*Disengage*					-.61	-8.38	<.001	[-2.03, -1.22]
*Reappraise*					-.41	-11.27	<.001	[-1.68, -1.16]
IFG→MFG	-.96	.26	.398	[-.63, .82]				
*Disengage*					-.88	.83	.209	[-.43, 1.01]
*Reappraise*					-.60	1.78	.045	[-.10, 1.23]
SMG→MFG	-.10	-2.20	.020	[-1.02, -.03]				
*Disengage*					-.05	-1.92	.034	[-1.15, .04]
*Reappraise*					.05	-1.70	.052	[-1.00, .10]
Input (Matrix C)								
V1	-.01	-1.17	.128	[-.02, .01]	.03	3.72	<.001	[.01, .04]

*Note.* Connectivity strength is expressed in Hertz (Hz). *t*- and *p*-values present the results of one-sample t-tests, which compared endogenous connectivity strengths with zero (Matrix A, C) and their modulated strength against the endogenous strength (Matrix B). *Abbreviations*: M = mean, CI = 95% confidence interval (only connectivity strengths with confidence intervals that do not cross zero are considered reliable), V1 = primary visual cortex, SMG = supramarginal gyrus, I/MFG = inferior/middle frontal gyrus, SMA = supplementary motor area.

Whilst Regulators selected an ER strategy for Targets during the Selection events, the endogenous excitatory signal from MFG to IFG (.79 [±.15] Hz) was modulated downwards (-.59 [±.22] Hz). In contrast, the endogenous inhibitory signal from IFG to MFG (-1.05 [±.15] Hz) was not modulated significantly. A similar pattern was observed in Targets when they implemented Regulator-selected ER strategies; the endogenous excitatory connection between MFG and IFG (1.02 [±.13] Hz) was down-modulated during both reappraisal (-.41 [±.13] Hz) and disengagement (-.61 [±.19] Hz), but the strength of the endogenous inhibitory signal from IFG to MFG (-1.16 [±.17] Hz) was not modulated significantly. Finally, in Regulators the endogenous excitatory connection between SMG and MFG (.42 [±.23] Hz) was down-modulated during their ER selections (-.10 [±.24] Hz), but the same endogenous connection (.50 [±.17] Hz) was not modulated significantly during Targets’ implementation of Regulator-selected strategies.

Since we did not counterbalance stimulus-responses mappings, Regulators always used their left thumb to select Disengage and their right thumb to select Reappraisal. For this reason, it was important to check that the connections and task-related modulations estimated by the bDCM applied to Selection events for Regulators were not driven purely by motor-related processes (e.g., executing left- or right-hand responses). Although Targets always used their right thumb when making their ratings, the bDCM was applied to the preceding 5-sec Implementation events when no motor response was required. As such, motor-related responses are unlikely to have influenced the bDCM applied to Targets. Supplementary Table S10 shows that no reliable correlations existed between parameters estimated by the bDCM and the sum of Regulators’ Disengage or Reappraisal selections, or Targets’ mean ratings (indexing the frequency of their right-thumb responses). This indicates that the model captured the cognitive and affective processes driving Regulators’ selections and Targets’ ratings rather than the resulting button presses.

### 3.4 Brain–behaviour relationships

Spearman correlations evaluated relationships among the degree to which effective connections among these nodes were modulated, self-reported personality characteristics (AQ, IRI, and ACS), and behavioural metrics of ER effectiveness (Prediction, Efficacy_*Inter*_, and Efficacy_*Intra*_). The full correlation matrices are presented in Supplementary Table S8 (Regulators) and S9 (Targets), and here we describe only significant and reliable correlations that inform the theoretical propositions outlined in the Introduction.

For Regulators, FS was correlated negatively with Efficacy*_Inter_* (*ρ*(21) = -.44, *p* = .043, CI = [-.68, -.08])*,* meaning that the strategies selected by Regulators with higher FS were *less* effective in down-regulating Targets’ emotional ratings. In terms of action orientation, AOF was correlated positively with Efficacy*_Inter_* (*ρ*(21) = .51, *p* = .016, CI = [.07, .81]) and the degree of down-modulation on the inhibitory IFG→MFG connection (*ρ*(21) = .43, *p* = .044, CI = [.02, .72]). Additionally, AOD was correlated positively with the down-modulation of the MFG→IFG connection (*ρ*(21) = .68, *p* < .001, CI = [.30, .87]). Greater action control in Regulators was, therefore, associated with more effective ER strategy selections during inter-personal ER and *less* strong inhibitory modulation of effective brain connections underpinning these selections. Finally, the *Prediction* metric was correlated positively with the down-modulation of the MFG→IFG (*ρ*(21) = .45, *p* = .035, CI = [.01, .79]) and the SMG→IFG connection (*ρ*(21) = .61, *p* = .003, CI = [.25, .83]). This indicates that higher accuracy in Regulators’ predictions of Targets’ upcoming emotional responses was associated with greater down-modulation of connectivity between these network nodes.

In Targets, AOD was associated negatively with the degree of modulation on the inhibitory IFG→MFG connection whilst Targets implemented Regulator-selected reappraisal (*ρ*(22) = -.48, *p* = .02, CI = [-.74, -.11]). Finally, the Efficacy*_Intra_* and Efficacy*_Inter_* metrics were correlated positively (*ρ*(22) = .87, *p* < .001, CI = [.65, .97]); in other words, the effectiveness of Regulator-recommended strategies was related strongly to Targets’ implementation of self-selected ER strategies. Sample personality characteristics are shown in Supplementary Table S7 and Figure S2.

## 4 Discussion

Decades of research has begun to define the cognitive and associated brain mechanisms that underpin intra-personal emotion regulation (ER; Morawetz et al., 2017, 2020), and how their disruption gives rise to dysfunctional self-directed ER. Very little is known about those supporting inter-personal ER, however, particularly the neurocognitive mechanisms underpinning our ability to regulate another person’s emotions. Since inter-personal ER promotes well-being in both the Regulator and Target (Cohen & Arbel, 2020; Guendelman et al., 2022), and given our proclivity to help others regulate their emotions in social situations (Liu et al., 2021; Tran et al., 2023), the present study addressed this important knowledge gap. To achieve this, we applied an input-state-output modelling technique (behavioural Dynamic Causal Modelling; bDCM) to functional brain imaging data acquired simultaneously from Regulators and Targets during inter-personal ER. This revealed that the recommendations made by Regulators and their effectiveness in down-regulating Targets’ negative emotional responses can be estimated with high accuracy from patterns of effective brain connectivity among five left-lateralised nodes: whilst some connections were modulated in Regulators when they selected a strategy for the Target, others were modulated in Targets when they implemented Regulator-selected strategies. These results provide new insights into the neurocognitive mechanisms supporting inter-personal ER.

By imaging the brains of both Regulators and Targets, we observed extensive overlap in the patterns of brain response elicited during the ER strategy selection and implementation stages of inter- and intra-personal ER, respectively. This overlap encompassed predominantly left-lateralised brain regions comprising ventro- and dorso-lateral prefrontal cortex (inferior and superior frontal gyrus, respectively; IFG and SFG), supplementary motor area (SMA), lateral temporal and temporo-parietal cortices (extending dorsally into the supramarginal gyrus; SMG). This finding is consistent with previous studies that have measured brain responses from both Regulators and Targets separately during inter- and intra-personal ER: during both Regulators’ attempts to influence a Target’s emotional responses and Targets’ implementation of predetermined ER strategies, Hallam et al. (2014) observed brain responses in the IFG and (pre-)SMA and Guendelman et al. (2022) report overlapping engagement of the left temporo-parietal cortex and precuneus—brain regions implicated in self-referential processing. Similarly, Xie et al. (2016) report the engagement of the Default Mode Network, comprising temporo-parietal cortex and precuneus, in Targets during their implementation of Regulator-selected ER strategies. Importantly, however, these earlier investigations focused on the implementation stage of the inter-personal ER process. The present study extends these findings by providing the first mapping of brain responses in Regulators whilst they select ER strategies to recommend for Targets. Interestingly, these brain responses occurred in regions implicated heavily in intra-personal ER (e.g., SMA; Morawetz et al., 2017, 2020), social cognitive processes such as social reasoning and self-other distinction (e.g., IFG, temporo-parietal cortex; Martin et al., 2020; Schurz et al., 2021; Van Overwalle, 2009), and cognitive (re)appraisal (Bo et al., 2024). Furthermore, brain responses did not differ between Regulators and Targets during their selection of ER strategies. Although the design of the current study did not permit direct (within-subject) comparisons between inter- and intra-personal ER for Regulators, these findings are consistent with existing evidence (He et al., 2025; Kozakevich Arbel et al., 2021) that understanding another’s affective experience relies on shared self and other neural representations and simulation processes. These shared mechanisms have been proposed to support inter-personal ER through embodied processes and (implicit) self-regulation (Barsalou et al., 2003; Guendelman et al., 2022; Winkielman et al., 2018).

Through bDCM, the present study has revealed patterns of effective connectivity among the SMA and left-lateralised SMG, IFG, and MFG that were modulated differently in Regulators and Targets, and this differential modulation could be used to accurately estimate Regulators’ strategy selections and the efficacy of their subsequent implementation by Targets during inter-personal ER. The high accuracy (90%) with which we estimated the efficacy of Regulator-recommended strategies in down-regulating Targets’ emotional responses from just the modulated connectivity among these brain regions provides further validation of the data-driven model proposed by Morawetz et al. (2016), and converges with other models of neural dynamics that have been associated with the efficacy (Bo et al., 2024) and habitual selection of reappraisal during intra-personal ER (Steward et al., 2021). However, it must be emphasised that these earlier neuroimaging data were acquired during the implementation stage of *intra*-personal ER. Our findings go further by demonstrating that a similar brain network supports *inter*-personal ER implementation, and neural signalling among its nodes can be used to accurately estimate the effectiveness of this process. Moreover, a similar pattern of neural connectivity also estimated Regulators’ selections with high accuracy (74%). These similarities in neural processing correspond with the overlap in brain systems implicated in explicitly (instructed) and implicitly (involuntarily) controlled intra-personal ER (Braunstein et al., 2017; Payer et al., 2012), which might interact during the inter-personal ER process.

The selection of ER strategies by Regulators and their implementation by Targets were both related to inhibitory modulations of the endogenous excitatory connection from the MFG to the IFG. Previous observations of a down-modulation in this dorsal-to-ventral prefrontal connection have been interpreted as an ongoing reappraisal of an emotion-eliciting stimulus, which is terminated by an inhibitory connection running in the reverse direction once a suitable strategy has been selected (Morawetz et al., 2016). A similar recursive appraisal process is likely to occur in Regulators; before selecting an ER strategy, they must attempt to appraise a stimulus from the Target’s perspective, consider their ER capabilities, and then evaluate the suitability, efficiency, and anticipated effort associated with different ER strategies (Matthews et al., 2022; Reeck et al., 2016). This initial evaluation might be guided by a process of embodied simulation associated with implicit intra-personal ER (Braunstein et al., 2017), particularly when Regulators have the few social cues that were available to them in the present paradigm (see Barsalou et al., 2003; Winkielman et al., 2018). Inhibitory connectivity from the MFG to IFG in Regulators could reflect the final integration of all these factors in the appraisal process and their ultimate selection of an ER strategy to recommend. It is important to acknowledge, however, that both Targets and Regulators had just two ER strategies from which to choose. Since the implementation of different ER strategies appears to involve varying levels of cognitive demand (Strauss et al., 2016) and might, therefore, rely on different patterns of brain connectivity, future research should expand the options of ER strategies to gain further insight into the neurocognitive mechanisms supporting inter-personal ER as it occurs in naturalistic settings.

In the model proposed by Reeck et al. (2016), the temporo-parietal cortex is assumed to play a key role in the social cognitive processes supporting inter-personal ER; mentalising about the Target’s aims and capabilities will guide Regulators’ selections (Matthews et al., 2022), and Targets must interpret the motivations behind Regulators’ recommendations in order to implement them as intended (Reeck et al., 2016). In partial support of this prediction, the present study revealed a modulation of the excitatory connection from the SMG to MFG in Regulators during their ER strategy selections and a relationship between this modulated connection and the accuracy of Regulators’ predictions of Targets’ ratings. This connection was not modulated during Targets’ implementation of Regulator-selected ER strategies, however, which might reflect the constrains imposed by our paradigm: unlike naturalistic inter-personal ER, whereby Targets must try to infer the motivations and intentions behind Regulators’ recommendations, the motivation of Regulators was defined explicitly at the start of this study.

The lack of reliable differences in Targets’ ratings during inter- and intra-personal ER in the current study converges with earlier reports of no discernible differences between these two forms of ER when conducted between strangers (Cannava & Bodie, 2017; Hallam et al., 2014; Zhang, Qiu, Tang, & Li, 2023), but differs from studies that report both immediate and lasting benefits of inter- compared with intra-personal ER between individuals in close relationships (e.g., Levy-Gigi & Shamay-Tsoory, 2017; Sahi et al., 2023). Future studies should examine whether and how relational factors influence the effects of inter-personal ER on the underpinning brain networks (Petrova & Gross, 2023). Simple adaptations of iERT paradigm that we describe below would make it well suited for assessing the influence of social factors on inter-personal ER.

Unlike previous studies of intra-personal ER (Bo et al., 2024), we observed no relationships between the various parameters of our model and behavioural metrics of ER effectiveness on the iERT (EfficacyInter and EfficacyIntra). To afford direct comparisons with earlier studies on intra- and inter-personal ER, these behavioural metrics were based on Targets’ self-reported intensity ratings. Although such subjective measures remain the standard operationalisation of ER efficacy in inter-personal ER research (Compas et al., 2023; Gratz et al., 2020; Meddeb et al., 2026), they are known to be influenced by factors such as self-awareness, context effects, and social desirability that might obfuscate relationships with other objective measures. To avoid this potential limitation, future studies should consider building on our study by adding implicit measures to quantify the effectiveness of intra- and inter-personal ER (e.g., Implicit Positive and Negative Affect Test; Quirin, Kazén & Kuhl, 2009). However, implicit measures of ER have been shown to have less reliability and predictive validity than subjective measures (for a review, see Corneille & Gawronski, 2024). Another alternative is physiological indices (e.g., electrodermal responses), but our own and others’ research has shown that such objective measures often diverge from subjective self-reports and might, therefore, capture a different stage of regulated emotional responses (Ngombe et al., 2022; Urry, 2009, 2010).

Interestingly, we found strong and reliable relationships between certain personality characteristics, iERT performance, and specific model parameters. In Regulators, higher FS was associated with *poorer* inter-personal ER efficacy. This unexpected relationship may reflect their excessive immersion in Target’s emotions (e.g., hyper-empathy); since successful inter-personal ER requires simultaneous regulation of one’s own and another’s emotions, increased emotional involvement will be counterproductive (Cohen & Arbel, 2020; Franklin-Gillette & Shamay-Tsoory, 2021; Gross, 2015; Guendelman et al., 2022). The degree of modulation on the MFG to IFG connection was associated positively with Regulators’ self-reported decision-related action control; that is, the ability to initiate actions, select between competing alternatives, and implement intentions quickly under demanding conditions. Since this connection is believed to be pivotal in selecting an ER strategy, it might serve as a mechanism through which highly action-oriented Regulators can exert more efficiency in the (implicit) down-regulation of their own emotional response to stimuli before considering ER strategies for Targets (Braunstein et al., 2017; Koole & Fockenberg, 2011). Furthermore, Regulators with higher action orientation appeared to select more efficacious strategies for their Targets, as shown by a positive relationship between Regulators’ action orientation and the effectiveness of interpersonal ER. In Targets, the modulated connection from the IFG to MFG during Reappraisal was related negatively with self-reported decision-related action control. As described above, this connection is proposed to terminate ER strategy selection in preparation for its implementation (Morawetz et al., 2016). Thus, Targets with a high decision-related action control might find it less cognitively effortful to find a way of reappraising an image when implementing this Regulator-selected ER strategy. This corresponds with the relationship between individual differences in reappraisal success during the down-regulation of negative emotions and increased connectivity between left IFG and dorsal prefrontal cortex reported recently (Morawetz et al., 2024). Future studies should match Regulator and Target dyads on these personality characteristics to assess more systematically how they influence inter-personal ER through these neurophysiological mechanisms.

### 4.1 Limitations and future directions

Our study introduces new ways of investigating the brain mechanisms supporting inter-personal ER; namely, applying behavioural DCM to functional brain imaging data acquired from Regulators whilst they select freely from ER strategies to recommend *and* from Targets whilst they implement these Regulator-selected strategies. Measuring brain responses of both individuals simultaneously whilst they co-created a unique inter-personal dynamic offers a more ecologically valid setting with which to investigate inter-personal ER as it occurs in the real world. However, certain aspects of the design we have employed present limitations that should be addressed in future research.

First, to assess relationships between Regulators’ dispositional empathic tendencies and their accuracy in predicting Targets’ emotional responses on Watch trials independently of any learning that might have occurred over preceding trials, we prevented Regulators from observing Targets’ intensity ratings. In doing so, Regulators were unable to evaluate the effectiveness of their recommendations and adapt accordingly. This created a unidirectional, closed-loop process that prevented us from capturing the mutually reciprocal and interdependent characteristic of naturalistic social interactions (Redcay & Schilbach, 2019). In other words, although our second-person paradigm provides a way of moving beyond the passive “spectator science” that has dominated social neuroscience (Schilbach et al., 2013), its implementation in the present study failed to fully embrace the potential of dual-brain scanning; we do not know whether similar results would be obtained from scanning the brains of Regulators or Targets sequentially whilst they performed the iERT with a non-scanned affiliate. Further, although it was possible for Regulators and Targets to learn which ER strategy their partner preferred across successive stimulus presentations, the randomised presentation of trial types precluded us from tracking if such learning had occurred. Previous studies have found that Regulators use feedback from Targets when recommending ER strategies over repeated interactions, and this appears to involve mentalising abilities (Kozakevich Arbel et al., 2021). Interestingly, such interpersonal adaptation appears to involve modulated effective connectivity among brain regions implicated in empathic expressions (i.e., anterior insula and anterior cingulate cortices; see Shaw et al., 2019). Future research that employs the iERT should, therefore, allow Regulators to receive feedback from Targets (e.g., observe their ratings after different ER strategy implementations), permitting the bidirectional and reciprocal characteristic of real-world inter-personal ER to emerge. This would enable the application of learning models to quantify the effects of such feedback on Regulators’ ER strategy selections and on brain systems supporting social inferences, and permit between-brain analyses to explore the reciprocal flow of information between interactants’ brains.

Second, to eliminate the confounding influence of social proximity on brain and behavioural measures of inter-personal ER, the present study investigated pairs comprising strangers who were equally unfamiliar with one another. Although our paradigm emulates inter-personal ER as it occurs naturally between two strangers, and evidence shows that strangers can regulate one another’s emotions effectively (Coan et al., 2006), inter-personal ER appears to be more effective among individuals in close relationships (Morawetz et al., 2021; Sahi et al., 2023). In our study, Regulators met Targets only very briefly before commencing the experiment and had only one cue (Targets’ choices of ER strategies across the recursive trials) on which to base their recommendations and predictions. In contrast, individuals in close relationships have previous interactions on which to base their recommendations. To achieve a more comprehensive understanding of interpersonal ER, future studies should investigate whether the findings of the present study extend to inter-personal ER between individuals in close relationships and when more direct social cues are available to Regulators and Targets. By providing brain and behavioural measures of inter-personal ER as a uni-directional process among unfamiliar strangers, the findings of the present study offer an important reference against which the effects of these important factors can be quantified.

Finally, because we never asked Regulators to self-select ER strategies for themselves to implement, we could not isolate neural responses involved specifically in inter-personal ER strategy selection by contrasting them directly with intra-personal ER strategy selection. As an alternative, we implemented the Frame condition as a control. Selecting a colour frame for someone else is motivationally and cognitively different from extrinsic ER strategy selection, however, and contrasting brain responses with this control condition may capture broader differences in goal states. Future studies should allow Regulators to alternate between selecting ER strategies for themselves and for Targets, allowing for self-other contrasts of ER strategy selection.

### 4.2 Conclusion

By applying an input-state-output modelling technique to behavioural and brain imaging data acquired simultaneously from Regulators and Targets during inter-personal ER, the present study provides the first insights into the neurocognitive mechanisms underpinning Regulators’ selection of ER strategies to recommend and their subsequent implementation by Targets. Both stages of this process were found to elicit responses in brain systems implicated in social cognition and cognitive control, and from similar patterns of effective connectivity among these brain systems, we could estimate with high accuracy the selections of Regulators’ and the effectiveness of their implementation by Targets. As implemented in the current study, however, the interpersonal Emotion Regulation Task captures only a specific type of inter-personal ER; namely, as it occurs between strangers with just two ER strategies to choose from, and when no explicit feedback is provided to Regulators about the effectiveness of their recommendations. As with any social process that occurs between two or more individuals, innumerable factors will exert interactive effects on the way that inter-personal ER unfolds in real-world settings. Since no single study can operationalise every possible combination of influencing variables, future research into inter-personal ER should systematically investigate the independent effects of different factors. Subsequent studies should examine whether and how the neurocognitive model supported by our data underlies inter-personal ER between individuals in close relationships, for example, and the modulating role of feedback in this social process. By investigating inter-personal ER as a unidirectional process among strangers, the findings of our study provide an important reference comparison in this endeavour.

## Supplementary Material

Supplementary Material

## Data Availability

All experimental materials (e.g., stimulus list), procedure and analysis codes, and behavioural and brain imaging data are available publicly at https://osf.io/8hmrq/.
