# Supplementary material for: A dual-fMRI investigation of interpersonal emotion regulation: Predicting strategy selection and implementation success from effective brain connectivity
Source: Imaging Neurosci (Camb). 2026 Sep 11;4:IMAG.a.1360. doi: 10.1162/IMAG.a.1360 (PMC13576947; doi:10.1162/IMAG.a.1360)
Supplement: Supplementary file 1 [file IMAG.a.1360_supp.pdf]

## Task Instructions

During the experiment, you and your partner will perform a simple task together.

At the very beginning of the experiment, you will be divided into roles. One of you will be assigned the role of a **"TARGET"** (**player A**) and the other will become the **"REGULATOR"** (**player B**). The assigned roles will not change during the course of the experiment.

Each round of the task consists of 5 parts. Throughout the task, both of you will be presented with the same stimuli at the same time. Each round starts with a cross placed in the centre of the screen. Please focus on this cross because it will be subsequently replaced by a very short presentation of a photograph (500 milliseconds). After the photo disappears from the screen and before it is presented again, you will be asked to complete one of the following tasks:

1. **Player A** will be asked to choose one of the two colours to frame the photo (blue or green).
2. **Player B** will be asked to choose one of the two colours to frame the photo (blue or green).
3. **Player A** will be asked to choose one of two strategies for regulating their emotions experienced while looking at the photograph.
4. **Player B** will be asked to recommend one of two strategies for regulating the emotions experienced by **player A** when they look at the photograph.
5. **Player B** will be asked to predict the emotional response of **Player A** in response to the photograph.

Afterwards, the same image will be presented again for 5 seconds and Player A will be asked to just look at it or to use the selected emotion regulation strategy. After these five seconds, Player A will be asked to rate his emotional response.

**Disengagement**
Try to make yourself feel less unpleasant when looking at the photo – think of something completely unrelated to the content of the photo. There are several ways to do this. First, you can imagine the surroundings of your house, or another well-known street. For example, if you look at a photo of a burned woman, you can think about riding a bike around campus and the various buildings around you. Second, you can imagine yourself performing everyday activities – like taking a shower or making coffee in the morning. You can use any of these ways to distract yourself from the photo that you feel will be most effective for you personally to make yourself feel less uncomfortable. You don't have to use the same way to distract or divert your attention throughout the experiment. However, it is very important for us that you keep looking at the photo throughout its entire presentation; your eyes should not be directed away from the photo. When disengaging from the photo, it is also important not to focus on something that is emotionally strong for you personally, because we also don't want you to think about something that elicits sadness or strong feelings of happiness.

**Reappraisal**
Try to make yourself feel less unpleasant when looking at the photo by changing its meaning. This means that you will think about something that will help you not to feel so uncomfortable. For example, you can think about an alternative outcome of the event you

are watching – e.g., the situation will be resolved soon or that help is on the way. It is also possible to focus on some detail in the photo that may not be as bad as it might seem at first glance. However, we want you to concentrate on the photograph the entire time it is presented on the screen and without thinking about random things that could help you feel better. Instead, the challenge is to modify something in your thinking about the content of the photo – something that will help you feel less unpleasant. Please focus on the photo throughout its presentation, but say something to yourself about it that will help you feel better when you look at it.

**Supplementary Table S1.** Normative ratings and arousal categorisation of IAPS stimuli.

| Stimuli | Valence | | Arousal | | |
|---|---|---|---|---|---|
| ID | M | SD | M | SD | low/high |
| 1301 | 3.70 | 1.66 | 5.77 | 2.18 | high |
| 2053 | 2.47 | 1.87 | 5.25 | 2.46 | low |
| 2100 | 3.85 | 1.99 | 4.53 | 2.57 | low |
| 2278 | 3.36 | 1.57 | 4.55 | 2.02 | low |
| 2312 | 3.71 | 1.64 | 4.02 | 1.66 | low |
| 2490 | 3.32 | 1.82 | 3.95 | 2.00 | low |
| 2682 | 3.69 | 1.65 | 4.48 | 2.10 | low |
| 2691 | 3.04 | 1.73 | 5.85 | 2.03 | high |
| 2800 | 1.78 | 1.14 | 5.49 | 2.11 | low |
| 3000 | 1.59 | 1.35 | 7.34 | 2.27 | high |
| 3068 | 1.80 | 1.56 | 6.77 | 2.49 | high |
| 3140 | 1.83 | 1.17 | 6.36 | 1.97 | high |
| 3150 | 2.26 | 1.57 | 6.55 | 2.20 | high |
| 3180 | 1.92 | 1.13 | 5.77 | 2.21 | high |
| 3230 | 2.02 | 1.30 | 5.41 | 2.21 | low |
| 3530 | 1.80 | 1.32 | 6.82 | 2.09 | high |
| 6010 | 3.73 | 1.98 | 3.95 | 1.87 | low |
| 6190 | 3.57 | 1.84 | 5.64 | 2.03 | low |
| 6836 | 3.46 | 1.61 | 5.47 | 1.91 | low |
| 7360 | 3.59 | 1.95 | 5.11 | 2.25 | low |
| 8485 | 2.73 | 1.62 | 6.46 | 2.10 | high |
| 9120 | 3.20 | 1.75 | 5.77 | 1.94 | high |
| 9181 | 2.26 | 1.85 | 5.39 | 2.41 | low |
| 9252 | 1.98 | 1.59 | 6.64 | 2.33 | high |

| | | | | | |
|---|---|---|---|---|---|
| 9405 | 1.83 | 1.17 | 6.08 | 2.40 | high |
| 9410 | 1.51 | 1.15 | 7.07 | 2.06 | high |
| 9420 | 2.31 | 1.59 | 5.69 | 2.28 | high |
| 9433 | 1.84 | 1.19 | 5.89 | 2.60 | high |
| 9440 | 3.67 | 1.86 | 4.55 | 2.02 | low |
| 9470 | 3.05 | 1.51 | 5.05 | 1.98 | low |

**Supplementary Table S2.** Linear Mixed Modelling process.

| Ratings | | | | | | | | | | |
|---|---|---|---|---|---|---|---|---|---|---|
| **Model Name** | **Simpler Comparison Model** | **Fixed Effects** | **Random Effects** | **Model Fit** | | | | **LRT Test** | | **Decision** |
| | | | | **AIC** | **BIC** | **LL** | **df** | **df** | **X2** | |
| Base Model<br>Random intercept only | - | - | Intercept | 11112 | 11130 | -5553.10 | 3 | | | |
| Model 1<br>Fixed Model no interactions | Base Model | Condition<br>Trial Type | Intercept | 11009 | 11044 | -5498.40 | 6 | 3 | 109.39*** | Keep Model 1, which has a significantly better fit. |
| Model 2<br>Fixed Model with Interactions | Model 1 | Condition<br>Trial Type<br>Condition x Trial Type | Intercept | 11012 | 11059 | -5498.00 | 8 | 2 | 0.96 | No significant difference, Keep the simpler model without interactions. |
| Model 3<br>Random Condition | Model 1 | Condition<br>Trial Type<br>Condition x Trial Type | Intercept<br>Condition | 11016 | 11075 | -5497.80 | 10 | 4 | 1.31 | Keep Model 1, as Model 3 could not be fit accurately possibly due to perfect collinearity amongst predictors. |
| Model 4<br>Random Trial Type | Model 1 | Condition<br>Trial Type<br>Condition x Trial Type | Intercept<br>Trial Type | 10998 | 11075 | -5486.00 | 13 | 7 | 24.83*** | Keep Model 4, which has a significantly better fit. |

| | | | | | | | | | | |
|---|---|---|---|---|---|---|---|---|---|---|
| Model 5<br>Random Condition and Trial Type | Model 4 | Condition<br>Trial Type<br>Condition x Trial Type | Intercept<br>Condition<br>Trial Type | 11005 | 11106 | -5485.60 | 17 | 4 | 0.87 | Keep Model 4, as Model 5 failed to converge. |

***Note*.** When modelling a factor as both a fixed and random effect improved model fit, this indicates that capturing individual variation provided a better explanation of the data beyond the fixed effect alone. Where there was no significant difference between the models, the simpler model with fewer degrees of freedom was carried forward for further comparisons.

## Arousal-level Analyses

Earlier studies have reported that reappraisal is preferred over disengagement in response to low-arousal negative images, but the reverse is true for high-arousal stimuli (e.g., Sheppes et al., 2014). In response to reviewer comments, we conducted the following additional analyses:

1. Comparisons between the distribution of ER strategy selections by Regulators ($Inter_{ER}$ trials) and Targets ($Intra_{ER}$ trials) in response to low- and high-arousal negative images (see Supplementary Table S3). This confirmed that both Regulators and Targets showed this same pattern of preference.
2. An additional LMM that included Arousal as a predictor of Targets' ratings (see Supplementary Table S4 and S5 below). No significant differences were observed in Targets' intensity ratings during their implementation of disengagement and reappraisal ER in response to low- or high-arousal images.

**Supplementary Table S3.** Distributions of ER strategy selections for low- and high-arousal negative images.

| | Disengagement | | Reappraisal | | Total |
|---|---|---|---|---|---|
| | Low | High | Low | High | |
| Regulator | 153 | 183 | 214 | 140 | 690 |
| Target | 166 | 182 | 202 | 140 | 690 |
| Total | 319 | 365 | 416 | 280 | |

***Note***: A preference was shown for disengagement over reappraisal in response to high-arousal images by both Regulators ($\chi^2(64) = 184.00$, $p < .001$) and Targets ($\chi^2(81) = 207.00$, $p < .001$), and for reappraisal over disengagement in response to low-arousal images by both Regulators ($\chi^2(49) = 152.38$, $p < .001$) and Targets ($\chi^2(64) = 184.00$, $p < .001$).

**Supplementary Table S4.** Linear Mixed Model applied to Targets' ratings as a function of Condition, Trial Type and Arousal.

| | Rating | | | | |
|---|---|---|---|---|---|
| *Predictors* | *Estimates* | *SE* | *95% CI* | *t* | *p* |
| (Intercept) | 4.51 | 0.21 | [4.10, 4.93] | 24.26 | **<.001** |
| Condition (Intra vs Inter) | -0.04 | 0.06 | [-0.17, 0.09] | 2622.07 | .530 |
| Trial Type (Frame vs Dis) | -0.77 | 0.15 | [-1.06, -0.48] | 40.47 | **<.001** |
| Tp (Frame vs Reap) | -1.16 | 0.14 | [-1.44, -0.88] | 62.22 | **<.001** |
| Arousal (High vs Low) | -1.69 | 0.14 | [-1.96, -1.42] | 35.76 | **<.001** |
| Trial Type (Frame vs Dis) × Arousal (High vs Low) | 0.45 | 0.16 | [0.14, 0.76] | 2632.21 | **.004** |
| Trial Type (Frame vs Reap) × Arousal (High vs Low) | 0.65 | 0.16 | [0.34, 0.96] | 2638.74 | **<.001** |
| **Random Effects** | | | | | |
| $\sigma^2$ | 2.74 | | | | |
| $\tau_{00}$ Pair | 0.93 | | | | |
| $\tau_{11}$ Pair.TrialTypeDis | 0.22 | | | | |
| $\tau_{11}$ Pair.TrialTypeReap | 0.12 | | | | |
| $\tau_{11}$ Pair.ArousalLow | 0.24 | | | | |
| $\rho_{01}$ | 0.06 | | | | |
| | -0.50 | | | | |
| | -0.87 | | | | |
| ICC | 0.19 | | | | |
| N Pair | 23 | | | | |
| Observations | 2695 | | | | |
| Marginal $R^2$ / Conditional $R^2$ | 0.161 / 0.323 | | | | |

***Note.*** Reference categories for the Condition, Trial Type, and Arousal effects were Intra$_{ER}$, Frame, and High, respectively. Model equation: Rating ~ 1 + (1 + Trial Type + Arousal | Pair) + Condition + Trial Type + Arousal + Trial Type: Arousal

**Supplementary Table S5.** Mean (±SD) of Targets' ratings in response to low- and high-arousal stimuli during Inter$_{ER}$, Intra$_{ER}$, Inter$_{Frame}$ and Intra$_{Frame}$ trials.

| **Condition** | **Trial Type** | **Arousal** | |
|---|---|---|---|
| | | Low | High |
| Inter | Disengagement | 2.37 (1.62) | 3.70 (1.91) |
| | Reappraisal | 2.33 (1.36) | 3.46 (2.00) |
| | Frame | 2.76 (1.68) | 4.48 (2.20) |
| Intra | Disengagement | 2.57 (1.59) | 3.79 (1.92) |
| | Reappraisal | 2.27 (1.31) | 3.26 (1.97) |
| | Frame | 2.87 (1.78) | 4.53 (2.19) |

***Note.*** Examination of the Trial Type-by-Arousal interaction revealed a significant difference between Frames and Disengagement trials during high arousal ($p < .001$), but not during low arousal ($p = .301$). Frame trials were rated significantly higher than Reappraisal trials for both high ($p < .001$) and low arousal images ($p = .003$). Moreover, no significant differences were observed in ratings between Disengagement and Reappraisal trials for either high ($p = .304$) or low arousal images ($p = .888$).

**Supplementary Table S6.** Coordinates of GLM localisations.

| Label | Selection | | | | | | | | | | Implementation | | | | | | | | | |
|---|---|---|---|---|---|---|---|---|---|---|---|---|---|---|---|---|---|---|---|---|
| | REGULATOR | | | | | TARGET | | | | | TARGET | | | | | | | | | |
| | *Inter$_{ER}$* **vs** *Inter$_{Frame}$* | | | | | *Intra$_{ER}$* **vs** *Intra$_{Frame}$* | | | | | *Inter$_{ER}$* **vs** *Inter$_{Frame}$* | | | | | *Intra$_{ER}$* **vs** *Intra$_{Frame}$* | | | | |
| | **x** | **y** | **z** | **Vol.** | ***t*** | **x** | **y** | **z** | **Vol.** | ***t*** | **x** | **y** | **z** | **Vol.** | ***t*** | **x** | **y** | **z** | **Vol.** | ***t*** |
| Frontal Pole | | | | | | | | | | | | | | | | -30 | 42 | 20 | 747 | 5.13 |
| | | | | | | | | | | | | | | | | -40 | 62 | 6 | 12 | 3.51 |
| | | | | | | | | | | | | | | | | 24 | 46 | 36 | 18 | 3.46 |
| SFG | -24 | 50 | 10 | 10377 | 6.23 | 0 | 12 | 58 | 14280 | 7.71 | | | | | | 10 | 14 | 54 | 3410 | 5.63 |
| | | | | | | 14 | 62 | 28 | 898 | 6.28 | | | | | | | | | | |
| MFG | -2 | 58 | 4 | 51 | 3.72 | | | | | | | | | | | -42 | 34 | 28 | 161 | 4.49 |
| IFG | -48 | 16 | 0 | 24 | 4.8 | 50 | 20 | -6 | 162 | 5.86 | | | | | | 50 | 16 | 2 | 1750 | 5.68 |
| Insula | | | | | | | | | | | | | | | | -32 | 20 | -6 | 652 | 5.46 |
| | | | | | | | | | | | | | | | | 28 | 24 | 8 | 32 | 4.45 |
| Pre. Gyrus | 0 | -20 | 64 | 16 | 3.41 | | | | | | | | | | | | | | | |
| Frontal Op. | | | | | | | | | | | | | | | | -28 | 24 | 12 | 68 | 4.28 |
| AG/SMG | -50 | -58 | 36 | 112 | 5.26 | -54 | -52 | 34 | 2370 | 7.58 | -38 | -60 | 26 | 1392 | 5.2 | 60 | -48 | 28 | 911 | 5.84 |
| Precuneus | -4 | -68 | 42 | 146 | 4.58 | | | | | | | | | | | | | | | |

| | | | | | | | | | | | | | | | | | | | | |
|---|---|---|---|---|---|---|---|---|---|---|---|---|---|---|---|---|---|---|---|---|
| | -6 | -56 | 62 | 12 | 3.37 | | | | | | | | | | | | | | | |
| Post. Gyrus | -48 | -20 | 22 | 51 | 5.06 | | | | | | | | | | | | | | | |
| SPL | -24 | -52 | 60 | 14 | 3.46 | | | | | | | | | | | | | | | |
| SMG/STS | -62 | -40 | 22 | 42 | 5.17 | | | | | | | | | | | | | | | |
| STG | -58 | -28 | 0 | 81 | 5.49 | | | | | | | | | | | -48 | -16 | -12 | 2349 | 6.06 |
| | | | | | | | | | | | | | | | | 54 | -24 | 0 | 190 | 5.73 |
| | | | | | | | | | | | | | | | | 64 | 10 | -14 | 38 | 4.39 |
| MTG | | | | | | -54 | -20 | -12 | 885 | 6.34 | -50 | -42 | 0 | 10 | 4.18 | | | | | |
| LOC | -38 | -64 | 28 | 14 | 4.17 | | | | | | | | | | | | | | | |
| ACC | 12 | 38 | 18 | 59 | 4.01 | | | | | | | | | | | | | | | |
| MCC | | | | | | | | | | | | | | | | 14 | 18 | 28 | 16 | 3.56 |
| PCC | -2 | -26 | 36 | 282 | 4.55 | | | | | | | | | | | | | | | |
| | 0 | -20 | 48 | 18 | 3.33 | | | | | | | | | | | | | | | |
| | -2 | -46 | 24 | 14 | 5.1 | | | | | | | | | | | | | | | |
| Pallidum | | | | | | | | | | | | | | | | -18 | 0 | -8 | 77 | 4.29 |
| Cerebellum | 50 | -56 | -30 | 89 | 6.27 | 44 | -70 | -32 | 2945 | 8.04 | | | | | | | | | | |

***Note***. Coordinates are provided in MNI space for significant clusters after non-parametric permutation testing ($p < .050$) with volumes exceeding 10 $mm^3$. Abbreviations: S/M/IFG = superior/middle/inferior frontal gyrus, Pre./Post. Gyrus = pre/postcentral gyrus, Frontal Op. = frontal operculum, AG = angular gyrus, SMG = supramarginal gyrus, SPL = superior parietal lobe, STS = superior temporal sulcus, S/MTG = superior/middle temporal gyrus, LOC = lateral occipital cortex, A/M/PCC = anterior/mid/posterior cingulate cortex.

**Supplementary Figure S1.** Model fit expressed as a difference in free energy between the bDCM and null model applied to each Regulator and Target.

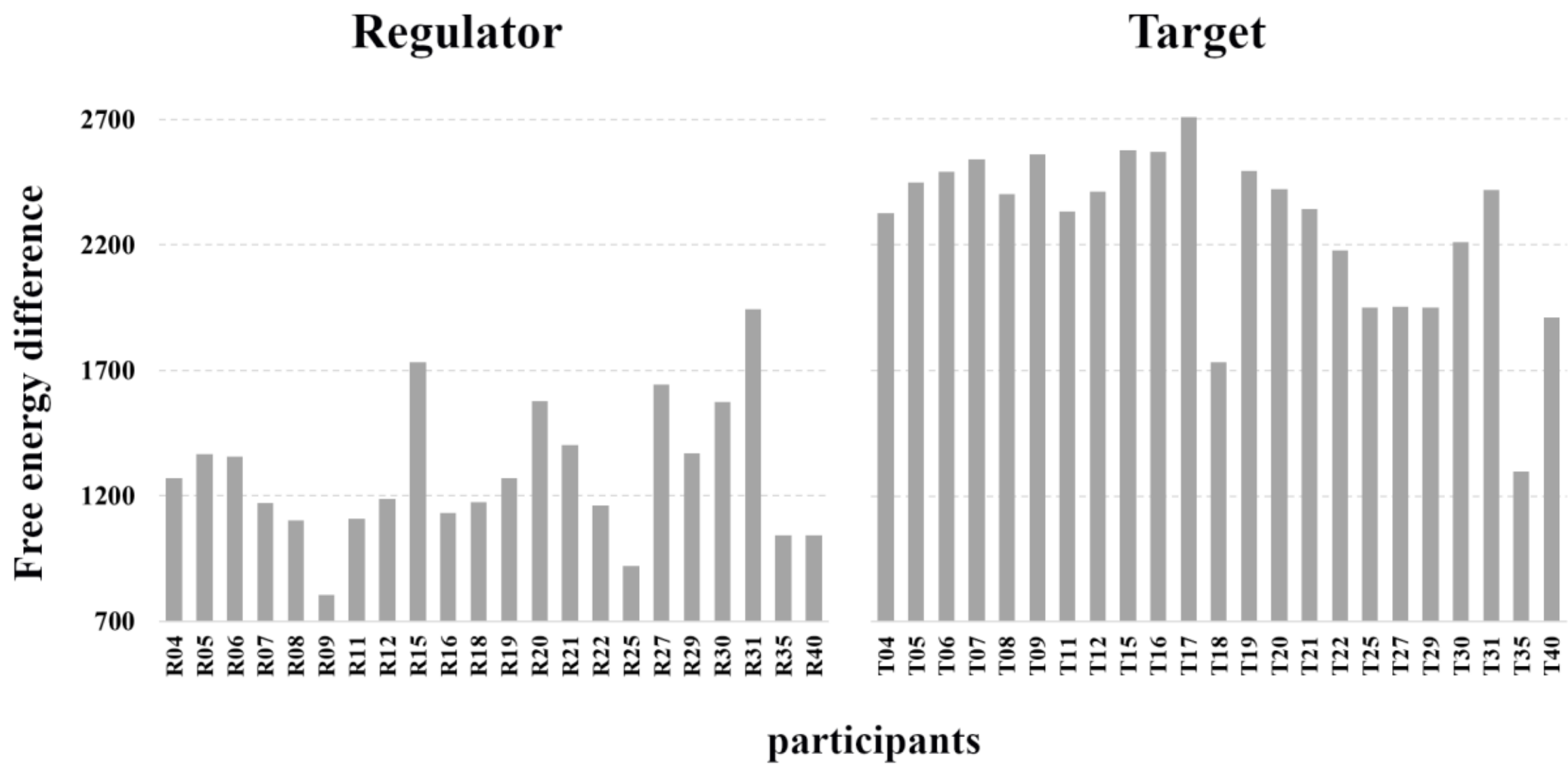


***Note*.** Free energy is an approximation of the model evidence that represents a compromise between model accuracy and model complexity to avoid bias towards over-parameterisation. Positive differences in free energy (*y*-axis) signify that the log model evidence of the bDCM model applied to individual Regulators (*left*) and Targets (*right*; *x*-axis) exceeded that from a null (trivial) model with a single node (vertex) and no directional connections (edges).

## Personality characteristics

Supplementary Table S7 presents the descriptive statistics for personality characteristics measured with the self-report instruments from both Regulators and Targets. Since pairs were not matched on these personality characteristics, all combinations were represented across the dyads (e.g., PT was higher in the Regulators of some pairs, but the reverse was true for others; see Supplementary Figure S2).

**Table S7.** Self-reported personality characteristics of the sample.

| | AQ | PT | FS | EC | PD | AOF | AOD |
|---|---|---|---|---|---|---|---|
| Regulators | 13(8) | 20(6) | 17(7) | 20(10) | 11(5) | 6(4) | 7(4) |
| Targets | 18(11) | 17(5) | 19(6) | 20(4) | 14(3) | 5(3) | 5(5) |

***Note.*** Median (and interquartile range) values are reported. AQ = Autism Spectrum Quotient, PT = perspective taking, FS = fantasy, EC = empathic concern, PD = personal distress, AOF = failure-related action-orientation, and AOD = decision-related action-orientation.

**Supplementary Figure S2.** Personality characteristics of Regulators and Targets across all pairs.

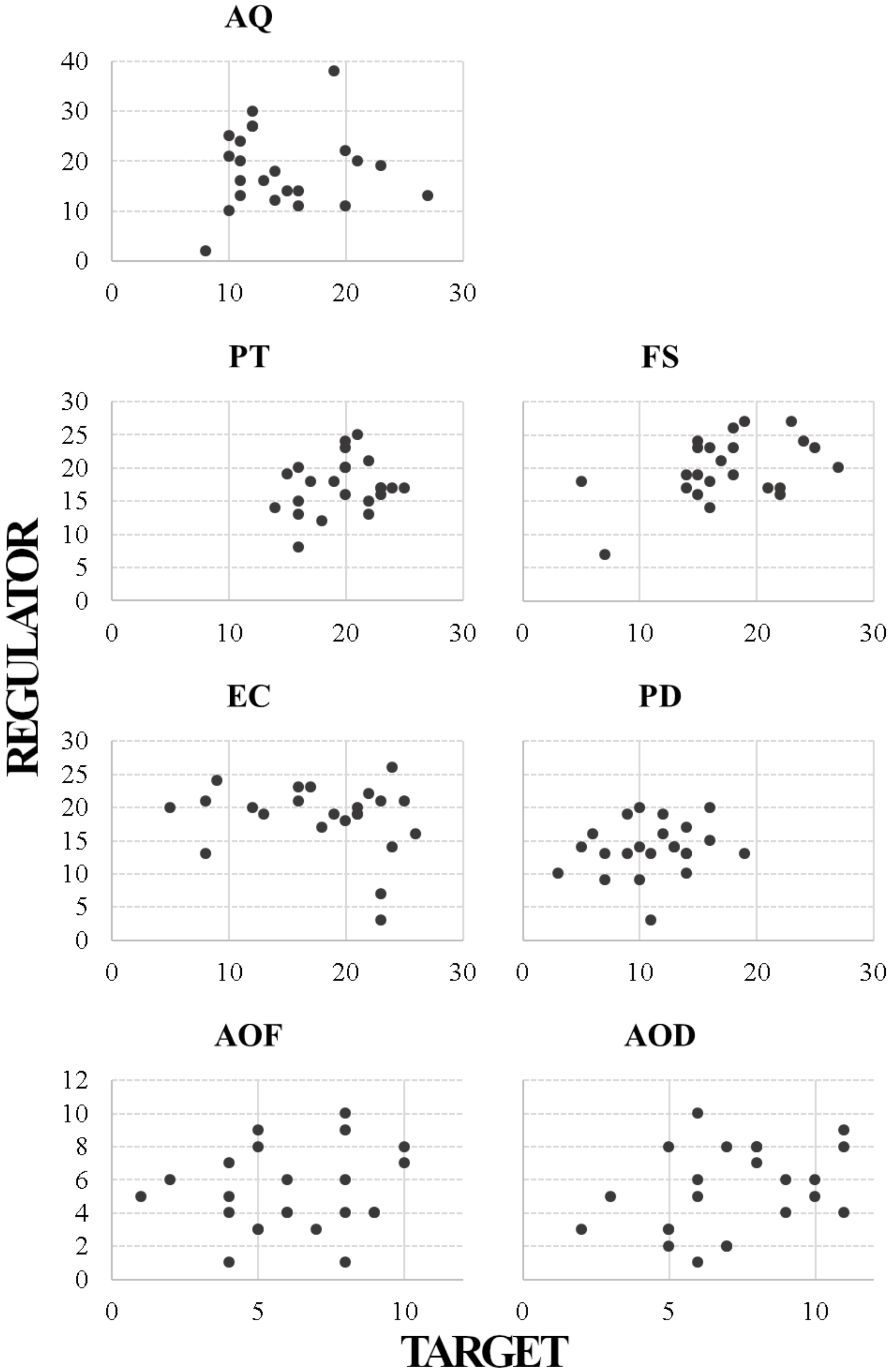


***Note.*** Plots present total scores for the Regulator and Target (*y*- and *x*-axis, respectively) comprising each pair for different sub-scales emerging from self-report measures. AQ = Autism Spectrum

Quotient, PT = perspective taking, FS = fantasy, EC = empathic concern, PD = personal distress, AOF = failure-related action-orientation, and AOD = decision-related action-orientation.

**Supplementary Table S8.** Associations between personality characteristics, behavioural metrics and brain responses for Regulators.

| | **AQ** | **PT** | **FS** | **EC** | **PD** | **AOF** | **AOD** | **SMG** | **MFG** | **IFG** | **SMA** | **MFG> IFG** | **IFG> MFG** | **SMG> MFG** | **Prediction** |
|---|---|---|---|---|---|---|---|---|---|---|---|---|---|---|---|
| **PT** | .07<br>[-.35, .49] | — | | | | | | | | | | | | | |
| **FS** | .11<br>[-.34, .51] | .22<br>[-.25, .62] | — | | | | | | | | | | | | |
| **EC** | -.22<br>[-.66, .25] | .31<br>[-.11, .66] | .19<br>[-.32, .60] | — | | | | | | | | | | | |
| **PD** | -.09<br>[-.63, .45] | -.09<br>[-.49, .35] | -.08<br>[-.48, .32] | .54**<br>[.13, .82] | — | | | | | | | | | | |
| **AOF** | -.13<br>[-.57, .32] | .15<br>[-.36, .59] | -.21<br>[-.57, .22] | -.30<br>[-.68, .14] | -.55**<br>[-.80, -.16] | — | | | | | | | | | |
| **AOD** | -.39<br>[-.73, .04] | .08<br>[-.37, .50] | -.11<br>[-.50, .27] | .21<br>[-.21, .58] | -.09<br>[-.52, .40] | .37<br>[-.08, .74] | — | | | | | | | | |
| **SMG** | .02<br>[-.45, .45] | .14<br>[-.38, .61] | -.25<br>[-.63, .21] | -.24<br>[-.61, .19] | -.49*<br>[-.79, -.08] | .40<br>[-.02, .77] | .00<br>[-.40, .41] | — | | | | | | | |
| **MFG** | .18<br>[-.28, .64] | -.05<br>[-.48, .44] | .08<br>[-.35, .49] | -.19<br>[-.56, .21] | -.32<br>[-.75, .10] | .15<br>[-.25, .59] | -.12<br>[-.60, .42] | .76**<br>[.54, .87] | — | | | | | | |
| **IFG** | .22<br>[-.20, .64] | .06<br>[-.40, .51] | .09<br>[-.35, .49] | .12<br>[-.32, .50] | .07<br>[-.38, .48] | -.17<br>[-.56, .32] | -.21<br>[-.63, .28] | .50*<br>[.13, .74] | .52*<br>[-.03, .90] | — | | | | | |
| **SMA** | .09<br>[-.40, .57] | .14<br>[-.31, .51] | -.19<br>[-.52, .20] | -.11<br>[-.50, .30] | -.22<br>[-.66, .26] | .03<br>[-.41, .54] | .10<br>[-.36, .54] | .62**<br>[.25, .85] | .39<br>[-.11, .73] | .64**<br>[.32, .81] | — | | | | |
| **MFG> IFG** | -.23<br>[-.68, .30] | -.03<br>[-.47, .42] | .23<br>[-.19, .59] | -.08<br>[-.54, .38] | -.18<br>[-.64, .39] | .31<br>[-.18, .71] | .68**<br>[.30, .87] | -.18<br>[-.53, .19] | -.16<br>[-.56, .34] | -.28<br>[-.66, .24] | -.01<br>[-.47, .46] | — | | | |
| **IFG> MFG** | -.37<br>[-.71, .03] | .04<br>[-.40, .45] | -.08<br>[-.53, .36] | -.11<br>[-.50, .36] | -.18<br>[-.60, .26] | .43*<br>[.02, .72] | .29<br>[-.14, .66] | .22<br>[-.15, .54] | .15<br>[-.32, .55] | -.14<br>[-.56, .30] | .12<br>[-.36, .55] | .39<br>[-.04, .73] | — | | |
| **SMG> MFG** | -.22<br>[-.55, .23] | -.19<br>[-.56, .19] | -.01<br>[-.43, .46] | -.06<br>[-.47, .34] | -.08<br>[-.52, .34] | .06<br>[-.39, .54] | .40<br>[.02, .70] | -.01<br>[-.41, .44] | .09<br>[-.41, .50] | .02<br>[-.42, .43] | -.03<br>[-.50, .43] | .59**<br>[.30, .76] | .46*<br>[-.04, .83] | — | |
| **Prediction** | -.43*<br>[-.74, .03] | -.13<br>[-.53, .35] | -.01<br>[-.43, .43] | -.10<br>[-.55, .37] | -.30<br>[-.67, .18] | .04<br>[-.46, .56] | .34<br>[-.15, .76] | .26<br>[-.16, .61] | .27<br>[-.21, .67] | .12<br>[-.38, .55] | .16<br>[-.36, .66] | .45*<br>[.01, .79] | .30<br>[-.14, .70] | .61**<br>[.25, .83] | — |
| **Efficacy *Inter*** | .01<br>[-.44, .46] | -.13<br>[-.52, .30] | -.44*<br>[-.68, -.08] | -.18<br>[-.64, .28] | -.18<br>[-.59, .32] | .51*<br>[.07, .81] | -.10<br>[-.53, .34] | .07<br>[-.37, .48] | -.23<br>[-.59, .20] | -.06<br>[-.50, .47] | -.05<br>[-.49, .49] | -.06<br>[-.47, .37] | -.15<br>[-.58, .32] | -.28<br>[-.67, .24] | -.17<br>[-.54, .33] |

***Note.*** Values in square brackets indicate the 95% confidence interval for each coefficient. * $p < .05$. **, $p < .01$. Although some correlations are significant, their confidence intervals indicate that the correlations are unreliable. AQ = Autism Spectrum Quotient, PT = perspective taking, FS = fantasy, EC = empathic concern, PD = personal distress, AOF = failure-related action-orientation, and AOD = decision-related action-orientation; these personality characteristics are expressed as self-reported ratings on Likert scales. SMG = supramarginal gyrus, MFG = middle frontal gyrus, IFG = inferior frontal gyrus; values represent correlations with z-values expressing the contrast Selection $Inter_{ER} > Inter_{Frame}$. > = endogenous directional (effective) connections between pairs of nodes on which Selection sub-trial events could have modulatory influences (expressed in Hertz). *Prediction* = mean absolute difference between Regulators' predictions and Targets' actual ratings on Watch trials. $Efficacy_{Inter}$ = mean difference between Targets' rating on $Inter_{ER}$ compared with $Inter_{Frame}$ trials.

**Supplementary Table S9.** Associations between personality characteristics, behavioural metrics and brain responses for Targets.

| | **AQ** | **PT** | **FS** | **EC** | **PD** | **AOF** | **AOD** | **SMG** | **MFG** | **IFG** | **SMA** | **MFG>IFG *Dis*** | **MFG>IFG *Reap*** | **IFG>MFG *Dis*** | **IFG>MFG *Reap*** | **SMG>MFG *Dis*** | **SMG>MFG *Reap*** | **Efficacy *Inter*** |
|---|---|---|---|---|---|---|---|---|---|---|---|---|---|---|---|---|---|---|
| **PT** | -.06<br>[-.47, .37] | — | | | | | | | | | | | | | | | | |
| **FS** | .19<br>[-.24, .57] | .10<br>[-.36, .54] | — | | | | | | | | | | | | | | | |
| **EC** | -.16<br>[-.48, .30] | .46*<br>[-.01, .79] | .24<br>[-.25, .66] | — | | | | | | | | | | | | | | |
| **PD** | .27<br>[-.24, .66] | -.08<br>[-.52, .40] | .21<br>[-.25, .62] | -.24<br>[-.58, .19] | — | | | | | | | | | | | | | |
| **AOF** | -.25<br>[-.62, .18] | .00<br>[-.46, .43] | -.02<br>[-.47, .47] | -.22<br>[-.57, .25] | -.37<br>[-.72, .10] | — | | | | | | | | | | | | |
| **AOD** | -.38<br>[-.69, .01] | .19<br>[-.26, .58] | -.21<br>[-.70, .32] | .08<br>[-.38, .52] | -.24<br>[-.63, .24] | .46*<br>[.03, .78] | — | | | | | | | | | | | |
| **SMG** | -.32<br>[-.70, .16] | .21<br>[-.30, .60] | .01<br>[-.45, .46] | -.34<br>[-.71, .11] | .10<br>[-.37, .56] | .04<br>[-.44, .47] | .01<br>[-.38, .40] | — | | | | | | | | | | |
| **MFG** | -.30<br>[-.65, .17] | -.04<br>[-.46, .42] | .12<br>[-.33, .47] | -.10<br>[-.55, .34] | .21<br>[-.23, .62] | -.03<br>[-.49, .43] | -.18<br>[-.57, .24] | .61**<br>[.20, .86] | — | | | | | | | | | |
| **IFG** | -.23<br>[-.59, .17] | -.05<br>[-.49, .35] | .04<br>[-.36, .40] | -.13<br>[-.57, .35] | -.27<br>[-.67, .15] | .24<br>[-.21, .60] | -.06<br>[-.45, .37] | .45*<br>[.01, .78] | .45*<br>[-.10, .79] | — | | | | | | | | |
| **SMA** | -.54**<br>[-.76, -.19] | .00<br>[-.40, .41] | -.03<br>[-.45, .39] | .04<br>[-.44, .46] | -.22<br>[-.57, .22] | .18<br>[-.22, .57] | .04<br>[-.40, .53] | .44*<br>[.02, .74] | .70**<br>[.29, .92] | .78**<br>[.50, .91] | — | | | | | | | |
| **MFG>IFG *Dis*** | -.08<br>[-.55, .44] | -.10<br>[-.50, .35] | -.24<br>[-.67, .26] | .21<br>[-.26, .62] | -.16<br>[-.56, .24] | -.11<br>[-.52, .33] | -.18<br>[-.62, .30] | -.18<br>[-.61, .30] | -.05<br>[-.48, .43] | -.02<br>[-.40, .36] | .02<br>[-.49, .46] | — | | | | | | |
| **MFG>IFG *Reap*** | -.05<br>[-.50, .42] | -.11<br>[-.56, .36] | -.31<br>[-.68, .12] | .16<br>[-.35, .62] | -.02<br>[-.49, .40] | -.18<br>[-.62, .31] | -.33<br>[-.69, .12] | -.11<br>[-.61, .37] | -.16<br>[-.68, .33] | -.20<br>[-.64, .29] | -.18<br>[-.72, .32] | .75**<br>[.49, .87] | — | | | | | |
| **IFG>MFG *Dis*** | -.10<br>[-.49, .28] | -.08<br>[-.53, .41] | -.10<br>[-.57, .35] | -.27<br>[-.65, .20] | .15<br>[-.34, .58] | .11<br>[-.31, .51] | -.07<br>[-.52, .39] | .17<br>[-.31, .62] | .24<br>[-.29, .73] | -.01<br>[-.47, .50] | .08<br>[-.39, .58] | .08<br>[-.40, .62] | -.04<br>[-.48, .39] | — | | | | |
| **IFG>MFG *Reap*** | -.25<br>[-.62, .23] | -.30<br>[-.64, .17] | -.11<br>[-.48, .29] | -.06<br>[-.50, .36] | -.07<br>[-.48, .42] | -.18<br>[-.63, .28] | -.48*<br>[-.74, -.11] | .10<br>[-.42, .57] | .20<br>[-.33, .59] | .24<br>[-.23, .60] | .31<br>[-.15, .61] | .37<br>[-.02, .67] | .47*<br>[.06, .77] | -.04<br>[-.45, .39] | — | | | |
| **SMG>MFG *Dis*** | -.03<br>[-.51, .43] | -.03<br>[-.46, .44] | -.01<br>[-.51, .49] | .37<br>[-.01, .72] | .08<br>[-.42, .54] | -.15<br>[-.52, .30] | -.10<br>[-.51, .34] | -.17<br>[-.59, .31] | .22<br>[-.29, .64] | .14<br>[-.36, .61] | .16<br>[-.33, .66] | .45*<br>[.00, .81] | .14<br>[-.32, .56] | -.23<br>[-.66, .29] | .06<br>[-.31, .46] | — | | |
| **SMG>MFG *Reap*** | -.11<br>[-.54, .38] | -.27<br>[-.62, .15] | -.21<br>[-.58, .25] | .07<br>[-.32, .46] | .04<br>[-.39, .49] | .06<br>[-.40, .52] | -.04<br>[-.44, .38] | -.21<br>[-.61, .26] | .16<br>[-.33, .55] | .15<br>[-.27, .53] | .26<br>[-.18, .60] | .25<br>[-.18, .63] | .01<br>[-.42, .47] | -.13<br>[-.54, .36] | .13<br>[-.40, .53] | .55**<br>[.17, .76] | — | |
| **Efficacy *Inter*** | -.25<br>[-.66, .23] | -.03<br>[-.44, .42] | -.15<br>[-.57, .29] | .08<br>[-.40, .51] | -.21<br>[-.60, .28] | -.05<br>[-.41, .35] | -.07<br>[-.48, .36] | -.04<br>[-.51, .41] | -.27<br>[-.68, .24] | .05<br>[-.42, .49] | .13<br>[-.33, .54] | .04<br>[-.38, .40] | .15<br>[-.29, .54] | -.24<br>[-.64, .24] | .15<br>[-.29, .53] | -.04<br>[-.49, .42] | .02<br>[-.42, .48] | — |
| **Efficacy *Intra*** | -.24<br>[-.69, .26] | -.11<br>[-.50, .33] | -.09<br>[-.53, .39] | -.03<br>[-.43, .37] | -.34<br>[-.67, .14] | .15<br>[-.31, .54] | -.07<br>[-.49, .40] | .07<br>[-.36, .49] | -.28<br>[-.69, .22] | .28<br>[-.19, .67] | .18<br>[-.32, .65] | .07<br>[-.36, .47] | .17<br>[-.24, .56] | -.19<br>[-.59, .32] | .21<br>[-.18, .56] | -.10<br>[-.55, .41] | -.15<br>[-.56, .32] | .87**<br>[.65, .97] |

***Note.*** Values in square brackets indicate the 95% confidence interval for each coefficient. * $p < .05$, **, $p < .01$. Although some correlations are significant, their confidence intervals indicate that the correlations are unreliable. AQ = Autism Spectrum Quotient, PT = perspective taking, FS = fantasy, EC = empathic concern, PD = personal distress, AOF = failure-related action-orientation, and AOD = decision-related action-orientation; these personality characteristics are expressed as self-reported ratings on Likert scales. SMG = supramarginal gyrus, MFG = middle frontal gyrus, and IFG = inferior frontal gyrus; values represent correlations with z-values expressing the contrast Implementation $Inter_{ER}$ > $Inter_{Frame}$. > = endogenous directional (effective) connections between pairs of nodes, on which the implementation of Regulator-selected Reappraisal (*Reap*) or Disengagement (*Dis*) could have modulatory influences (expressed in Hertz). $Efficacy_{Inter}$ = the mean difference between Targets' ratings on $Inter_{ER}$ compared with $Inter_{Frame}$ trials. $Efficacy_{Intra}$ = the mean difference between Targets' ratings on $Intra_{ER}$ compared with $Intra_{Frame}$ trials.

**Supplementary Table S10.** Correlations between BOLD responses, bDCM parameters and the frequency of motor responses.

| | **REGULATORS** | | | **TARGETS** | |
|---|---|---|---|---|---|
| | Disengage | Reappraise | | Disengage | Reappraise |
| | | GLM: $Inter_{ER} > Inter_{Frame}$ | | | |
| **IFG** | .05 [-.41, .51] | .30 [-.07, .59] | **IFG** | .02 [-.44, .46] | .21 [-.24, .60] |
| **MFG** | -.07 [-.52, .41] | .15 [-.29, .55] | **MFG** | -.25 [-.67, .24] | -.20 [-.60, .26] |
| **SMG** | -.34 [-.69, .13] | .22 [-.24, .62] | **SMG** | -.16 [-.59, .35] | .18 [-.21, .52] |
| **SMA** | -.29 [-.66, .24] | .27 [-.24, .67] | **SMA** | .05 [-.42, .45] | .17 [-.26, .59] |
| | | bDCM: Endogenous connectivity (Matrix A) | | | |
| **V1>SMG** | -.25 [-.63, .22] | .27 [-.16, .64] | **V1>SMG** | .00 [-.47, .48] | -.11 [-.57, .41] |
| **SMG>MFG** | -.03 [-.49, .43] | -.11 [-.61, .32] | **SMG>MFG** | -.45* [-.78, .00] | -.31 [-.64, .14] |
| **MFG>IFG** | -.04 [-.44, .43] | -.25 [-.64, .19] | **MFG>IFG** | -.03 [-.43, .37] | .13 [-.34, .54] |
| **MFG>SMA** | .02 [-.43, .47] | -.44* [-.74, .02] | **MFG>SMA** | .07 [-.37, .47] | .09 [-.37, .52] |
| **IFG>MFG** | -.05 [-.47, .36] | -.13 [-.54, .36] | **IFG>MFG** | .36 [-.08, .72] | .14 [-.30, .59] |
| **SMA>MFG** | .31 [-.14, .68] | -.24 [-.65, .24] | **SMA>MFG** | .19 [-.36, .64] | .24 [-.23, .62] |
| | | bDCM: Modulation (Matrix B) | | | |
| **MFG>IFG** | .19 [-.33, .62] | .13 [-.35, .58] | **MFG>IFG *Dis*** | -.03 [-.45, .42] | -.25 [-.57, .18] |
| **IFG>MFG** | -.02 [-.42, .39] | -.04 [-.45, .36] | **IFG>MFG *Dis*** | -.34 [-.71, .16] | -.18 [-.57, .31] |
| **SMG>MFG** | .18 [-.24, .54] | .21 [-.25, .61] | **MFG>IFG *Reap*** | .11 [-.30, .53] | -.05 [-.45, .39] |
| | | | **IFG>MFG *Reap*** | .13 [-.33, .57] | .07 [-.29, .41] |
| | | | **SMG>MFG *Dis*** | .07 [-.40, .53] | -.28 [-.68, .21] |
| | | | **SMG>MFG *Reap*** | -.03 [-.51, .46] | -.04 [-.46, .40] |

***Note.*** Values present Spearman correlations (with 95% confidence intervals). For Regulators (*left*), the sum of Disengage or Reappraise selections (left- and right-hand responses, respectively) on $\text{Inter}_{ER}$ trials were correlated with mean parameter estimates expressing the GLM contrast Selection $\text{Inter}_{ER}$ > $\text{Inter}_{Frame}$ from network nodes (spheres of 5 mm), and endogenous effective connections (A Matrix) and their task-dependent modulation (Matrix B) estimated by the bDCM. For Targets (*right*), the ratings they provided (higher values indexing more right-hand responses) in response to Regulator-selected Disengage or Reappraise on $\text{Inter}_{ER}$ trials were correlated with mean parameter estimates expressing the Implementation $\text{Inter}_{ER}$ > $\text{Inter}_{Frame}$ contrast from the same network nodes, and endogenous effective connections and their recommendation-dependent modulation estimated by the bDCM. * = $p < .05$, but the corresponding confidence intervals indicate that these results are unreliable; IFG = inferior frontal gyrus; MFG = middle frontal gyrus; SMG = supramarginal gyrus, SMA = supplementary motor area; > = endogenous directional (effective) connectivity.

## Habituation Effects

Since each of the 30 negative images were presented to participants five times—once in each condition—we investigated if the brain responses of Regulators or Targets demonstrated any evidence of a habituation effect that might have influenced the selection and/or subsequent implementation of ER strategies. To achieve this, we examined if a linear or step-like decrease occurred in BOLD responses over successive stimulus presentations during the initial Preview sub-trial event: we created five regressors comprising the Preview onsets of the first, second, third, fourth or fifth instance of each image. The Fixation, Choice, Preparation, Implementation and Rating sub-trial events were modelled as events of no-interest. At the participant level, we then performed fixed-effects GLM on these regressors with FEAT to capture linear decreases in BOLD signal across the five successive image presentations [2 1 0 –1 –2] and a step-like decrease in BOLD signal after the first image presentation [1 -.25 -.25 -.25 -.25]. Contrast estimates from these first-level analyses were carried forward for group-level analyses where they were evaluated with mixed-effects GLM and cluster-based thresholded at z>3.0, p<.05 (see Supplementary Figure S3).

**Supplementary Figure S3.** Habituation effects.

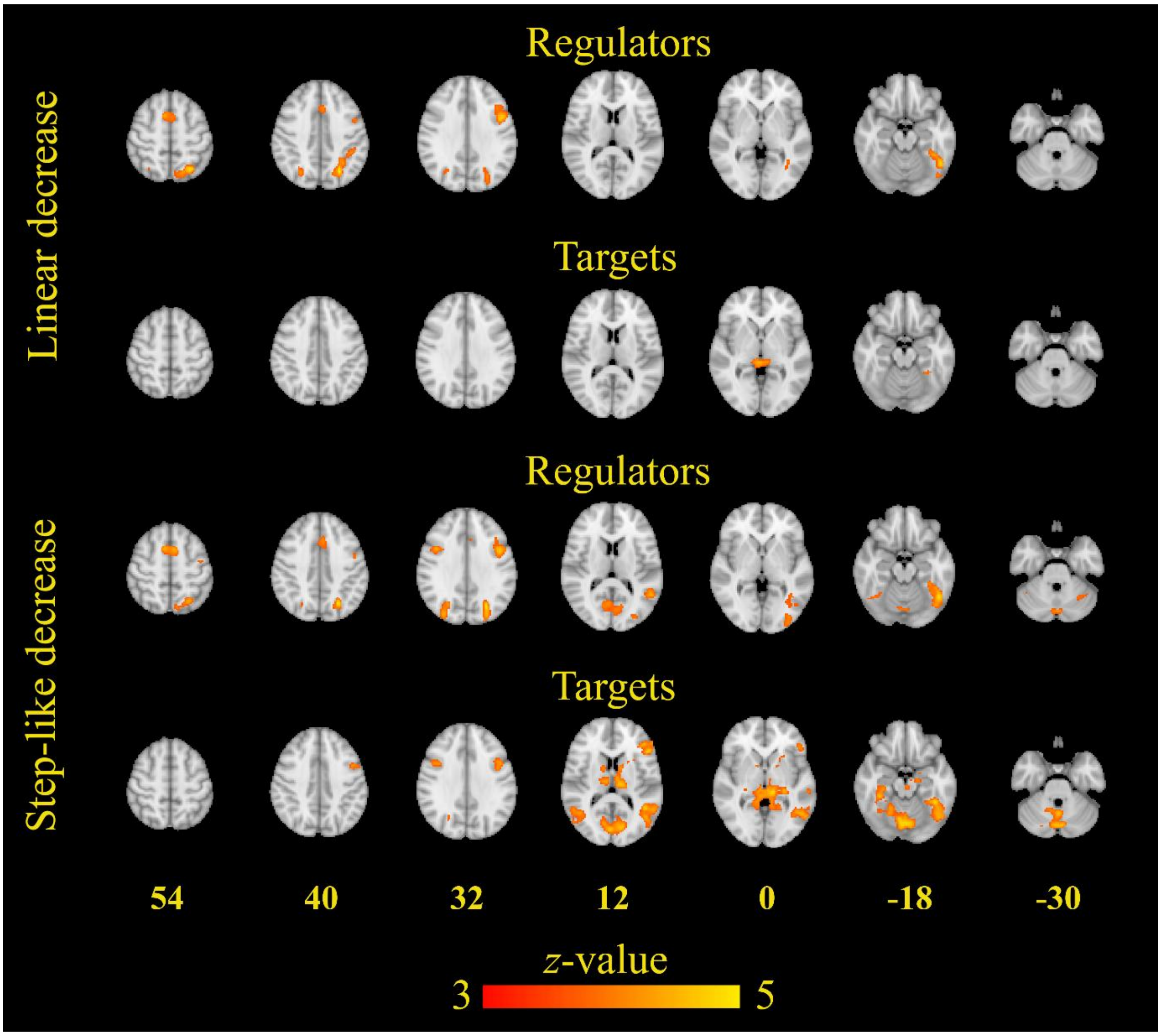


***Note.*** Values at the bottom present z-axis co-ordinates (MNI space).
